# Biochar filtration of drug-resistant bacteria and active pharmaceutical ingredients to combat antimicrobial resistance

**DOI:** 10.1038/s41598-024-83825-2

**Published:** 2025-01-08

**Authors:** Paul-Enguerrand Fady, Alexandra K. Richardson, Leon P. Barron, A. James Mason, Roberto Volpe, Meredith R. Barr

**Affiliations:** 1 Institute of Pharmaceutical Science, School of Cancer & Pharmaceutical Sciences, Faculty of Life Sciences & Medicine, King’s College London, 150 Stamford Street, London, SE1 9NH UK; 2 MRC Centre for Environment and Health, Environmental Research Group, School of Public Health, Imperial College London, 86 Wood Lane, London, W12 0BZ UK; 3 Department of Analytical, Environmental & Forensic Sciences, Institute of Pharmaceutical Science, School of Cancer & Pharmaceutical Sciences, Faculty of Life Sciences & Medicine, King’s College London, 150 Stamford Street, London, SE1 9NH UK; 4 School of Engineering and Materials Science, Queen Mary University of London, Mile End Road, London, E1 4NS UK; 5 Division of Chemical & Energy Engineering, School of Engineering, London South Bank University, 103 Borough Rd, London, SE1 0AA UK; 6 Department of Aeronautics, Faculty of Engineering, Imperial College London, Exhibition Rd, London, SW7 2AZ UK; 7 Present Address: Biosecurity Policy Unit, The Centre for Long-Term Resilience, 71-75 Shelton Street, London, WC2H 9JQ UK

**Keywords:** Wastewater treatment, Human pathogens, Pharmapollution, Pyrolysis, Morphology, Adsorption, Chemical engineering, Pollution remediation, Mass spectrometry, Environmental biotechnology, Antimicrobial resistance, Bacteria, Environmental microbiology, Crop waste

## Abstract

Antimicrobial resistance (AMR) is a major cause of death worldwide, with 1.27 M direct deaths from bacterial drug-resistant infections as of 2019. Dissemination of multidrug-resistant (MDR) bacteria in the environment, in conjunction with pharmapollution by active pharmaceutical ingredients (APIs), create and foster an environmental reservoir of AMR. Creative solutions are required to mitigate environmental AMR, while taking into consideration other aspects of the planetary “Triple Crisis” of pollution, biodiversity loss, and climate change. Waste lignocellulosic biomass (LCB), a byproduct of agriculture and forestry, is the largest stream of non-edible biomass globally. Through pyrolysis, waste LCB can be converted into biochars, which have excellent attributes for adsorption of pollutants–though no studies have yet reliably correlated production conditions with efficacy, nor considered adsorption of human pathogens. By leveraging a bespoke pyrolysis reactor with precisely controlled parameters, we show that production conditions substantially affect sequestration of clinical bacterial isolates, removing up to 94% of *Pseudomonas aeruginosa* RP73 and 85% of *Staphylococcus aureus* EMRSA-15. In addition, we show that chars produced at higher peak pyrolysis temperatures (450 °C) can remove up to 88% of the antibiotic clarithromycin from wastewater, as well as significant proportions of many other APIs with varied physicochemical characteristics. These findings provide a first-in-kind insight into how production conditions affect the ability of biochars to mitigate environmental AMR.

**Supplementary Information:**

The online version contains supplementary material available at 10.1038/s41598-024-83825-2.

## Introduction

Antimicrobial resistance (AMR) is a naturally-arising evolutionary phenomenon whereby micro-organisms develop the ability to tolerate compounds which would usually kill them or inhibit their growth^1,2^. Infections by bacteria resistant to clinical antibiotics were directly responsible for 1.27 million deaths per year globally as of 2019, and associated with 4.95 million deaths^3^. According to the projections of a report commissioned by the UK Government, up to 10,000,000 people annually could die from resistant infections globally by 2050, with a cumulative economic loss of 100 trillion USD if nothing is done to curb AMR^4^. The World Health Organisation considers preventative measures of controlling AMR, such as hygiene, vaccination, and sanitation (wastewater and sewage treatment) equally as important as antibiotic drug development–if not more so^5^. Therefore, creative, non-therapeutic solutions upstream of the clinic are urgently needed^6^.

The work herein demonstrates the development of non-pharmaceutical AMR mitigation solutions in a sustainable manner through the pyrolysis of waste lignocellulosic biomass (LCB). This is the first study to both use extremely precise pyrolysis conditions for agricultural waste biomass and show that these yield appropriate materials for effective in-line filters that sequester multi-drug-resistant (MDR) bacteria, as well as active pharmaceutical ingredients (APIs). The strains used in this work are clinical isolates of high priority human pathogens (per the World Health Organization), improving the real-world relevance of this work relative to previous studies in this field. This work is also unique in that it shows that a reduction in both resistant organisms and the concentration of selective and co-selective pharmapollutants in a real wastewater matrix is possible. No studies to date have brought together clinical isolates of human pathogens, tandem mass spectrometry of APIs, electron microscopy, and precise production conditions of well-characterised feedstocks. This will allow further development of upstream solutions which are critical to curtailing the prevalence of AMR in the environment and reducing the number of resistant infections globally.

The emergence of AMR in the environment poses the most substantial AMR-related health and safety risk for humans^7^, aside from the overuse and misuse of clinical antimicrobials, which are actively being addressed through a number of antimicrobial stewardship efforts^8–10^. Wastewater plays a major role in environmental AMR development. According to the World Water Assessment Programme (United Nations)^11^, 80% of wastewater is discharged into the environment untreated each year. In England, there are approximately 15,000 sewage overflow (SO) points across the country, of which 89% directly discharge into freshwater bodies^12^. During 2023, there were 464,056 spill events from all monitored SOs in England and Wales, amounting to 3,606,170 h of discharge in a single calendar year^13^. This has resulted in measurable concentrations of several antibiotics in British rivers for many years^14^.

Hospital and pharmaceutical manufacturing plant effluents are a major source of antimicrobial pollution (with the former also releasing resistant pathogens) which contributes to selective pressures in the environment and further develops AMR^15–20^. Proper treatment of wastewater (particularly from hospitals and the pharmaceutical industry) has been identified as a key strategy to helping prevent the spread of AMR^21–25^.

Current wastewater treatment plant (WWTP) technologies alone are inadequate for reducing environmental AMR, even during optimal operation and when not engaging in “bypass” (the release of untreated sewage into the environment)^26^. WWTPs are known contributors to environmental AMR, as they allow flows from various sources to mix, creating a “hotspot” for horizontal gene transfer between resistant and non-resistant bacteria^27,28^. Even when they are successful in reducing absolute bacterial loads, WWTPs can select for the most resistant bacteria^18^. Likewise, WWTPs do not effectively remove antimicrobial compounds from the waste stream^29–31^. In the River Wandle, located in the southeast of London, the antibiotics azithromycin, clarithromycin, sulfamethoxazole, and sulphapyridine have been detected at concentrations ranging from 132 ng*/*L to 476 ng*/*L directly downstream of the outfall from the local WWTP^32^.

A potential solution to this problem is the filtration of clinical and pharmaceutical wastewater prior to its arrival at WWTPs. Such in-line filters would remove bacteria and antimicrobials upstream of WWTPs. This would have two benefits: preventing horizontal AMR gene transfer at WWTPs, which act as key interfaces between non-resistant and resistant bacteria;^33^ and reducing total prevalence of ARGs and ARBs in WWTPs, so that fewer are released into the environment during a CSO or bypass event. To minimise environmental impact from manufacture and promote sustainable practices, these filters should be produced from waste materials using mild process conditions, as is the case for waste lignocellulosic biochars. Filters installed in e.g. sink traps, shower drains, or batch-production waste lines additionally benefit from repeated wet-dry cycles, which have been shown to augment the bacterial-retention capacity of biochars^34^. However, it is important to consider the possibility of developing resistance within such filters. While the mechanisms of resistance to adsorption itself would not be expected to drive cross-resistance to clinical antimicrobials, exposure to APIs in close proximity might. Therefore, it is critical to consider how spent filters, bearing a substantial load of hazardous substances, might be safely be treated to enable safe disposal and/or reuse. One possibility is re-pyrolysis, i.e. renewed exposure of the filter to heat without addition of an oxidising atmosphere. During this process, the elevated temperature would be expected to kill adsorbed microorganisms and degrade adsorbed APIs. This would also increase the surface area available for adsorption, which may have diminished as a result of extended use.

Biochars are the thermal or thermochemical decomposition products of LCB. LCB is the largest stream of non-edible biomass globally, the primary sources of which are agricultural and forestry waste streams^35^. This work focuses on walnut shells, a food systems by-product with a high surface area cell structure^36^. While LCB is naturally porous, pyrolysis and gasification have been shown to vastly increase its porosity^37–41^, and therefore the surface area available for adsorption. Biochars are widely used for a variety of adsorption-dependent environmental remediation applications including air, water, and soil treatment^42^.

With respect to bacteria, biochars are most commonly used for soil bioremediation using bacteria-impregnated^42–46^, or virgin biochars^42,47–53^ There is therefore a knowledge gap around the ability of biochars to sequester bacteria or APIs for the treatment of wastewater, which this work seeks to address. Previous studies on the sequestration of bacteria by biochars (herein referred to as bacterial adsorption) have largely used biochars as additives to conventional wastewater filters,^34,54,55^ or to soil^47,50,51^ A few have tested bacterial adsorption directly in liquid medium using a batch process^48,56^, but none have tested the ability of biochars alone to adsorb and retain bacteria in-line, as would be the case in sink trap/shower drain filters. The primary shortcoming of previous studies of bacterial adsorption has been the lack of control over pyrolysis conditions. Existing studies of bacterial adsorption using biochars either do not specify key pyrolysis process conditions, such as atmosphere, peak temperature, heating rate, or residence time at peak temperature,^47,50,54,54^ or specify broad ranges of these values, implying highly heterogeneous samples^34,50,51,55^ In addition, none of these studies specify more sophisticated process conditions, such as gas flowrate, detailed reactor design, or sample size & configuration. Reactor design and process parameters in biomass pyrolysis experiments have been shown to dramatically affect pyrolysis outcomes^57^. There have been some attempts to link pyrolysis peak temperature to bacterial adsorption properties^50,51,54^, but without reactor and experimental design allowing for the repeatable production of homogeneous biochar samples, the reliability of these results is inconclusive. There is therefore a clear knowledge gap around how these conditions affect adsorption efficacy, which this work further seeks to address.

Another shortcoming of existing studies is the use of non-clinically-relevant strains of bacteria. Only one of the aforementioned studies (Naka et al.^56^) tested adsorption of human pathogens (*E. coli* O157:H7). This study used “commercially-obtained activated charcoal” for which no production conditions were specified. No existing publications report testing those species most often associated with antibiotic resistance (“ESKAPE pathogens”)^58^, or any species on the WHO’s “bacterial priority pathogens list” such as drug-resistant *Pseudomonas aeruginosa* or *Staphylococcus aureus*^59^. There is therefore a clear knowledge gap around the efficacy of biochars to sequester the types of bacteria most relevant to human health and disease, which this works seeks to address.

The removal of APIs from waste streams using biochar adsorbents is an attractive strategy due to the cost-effective and sustainable nature of the product compared to existing solutions^60,61^ Antibiotic removal using biochar in particular has been extensively characterised in the literature, with some studies reporting almost 100% removal of compounds using a variety of biochar feedstocks.^63,61^ However, most studies have characterised the adsorption of APIs from synthetic wastewater or other aqueous matrices^64^ at concentrations over 20-fold greater than what is typically observed in influent wastewater (less than 700 ng/L)^65^. This is not necessarily representative of the complexity of a true wastewater matrix and the concentrations of APIs present in real-world samples that may affect adsorption. There is therefore a knowledge gap around the API adsorption efficacy of biochars when these compounds are in true wastewater matrices, which this work seeks to address.

This study presents an optimised workflow for accurately testing the effects of pyrolysis conditions and resulting biochar morphologies on bacterial and API adsorption performance (measured as the percentage of a particular contaminant sequestered upon filtration through a bed of biochar). The primary objective of this work was to determine the optimal biochar production conditions for the removal of Gram-negative and Gram-positive critical and high-priority ESKAPE pathogens and APIs from a wastewater matrix. Biochar morphologies were characterised using a synchrotron X-ray microtomography and in-situ radiography, as described in previous work^37^. This work demonstrates that the in-line sequestration of APIs and clinical strains of resistant pathogens using biochars produced from walnut shells is feasible, and that morphology & adsorption performance of these biochars may be tailored by choice of production conditions.

## Materials and methods

**Fig. 1:**
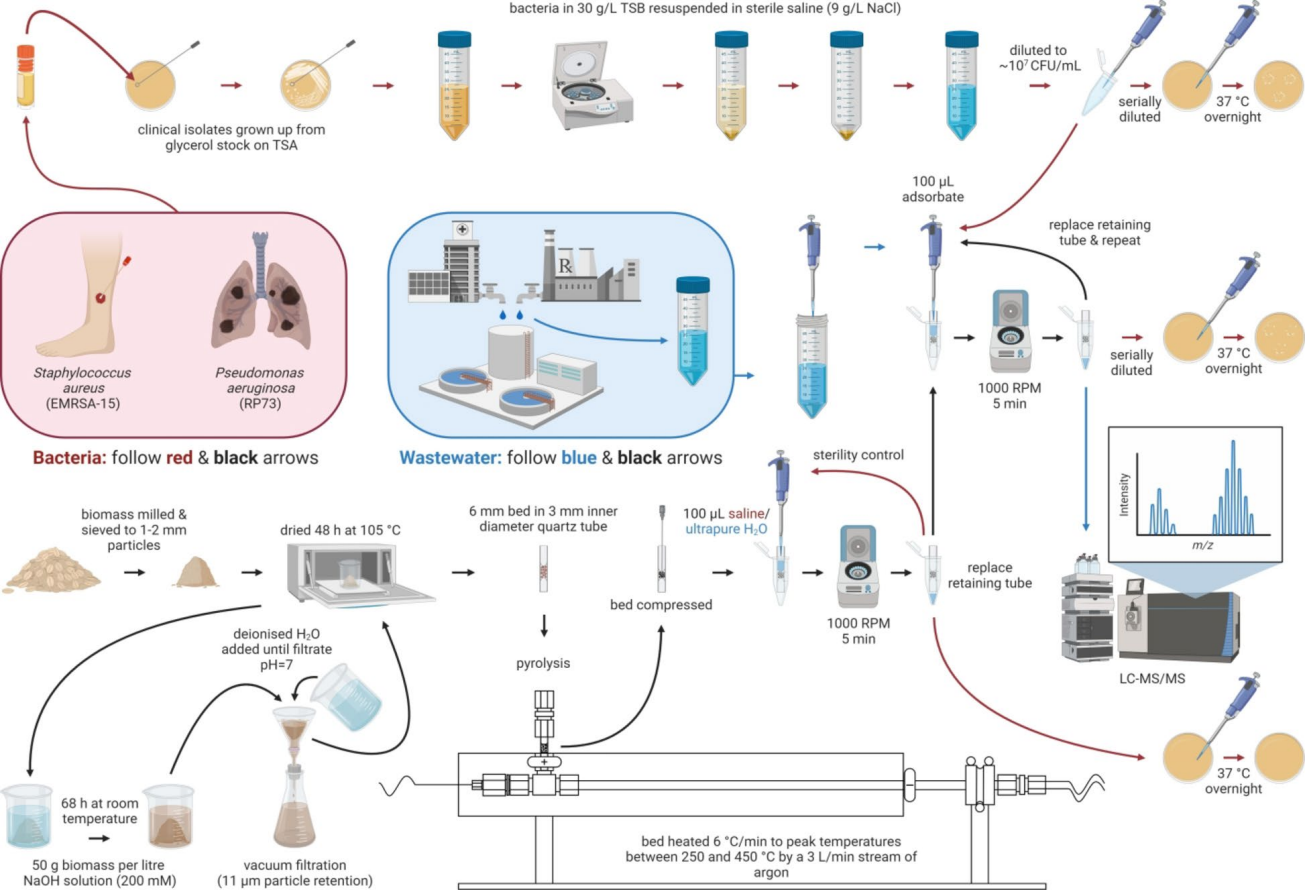
Graphical workflow of filtration experiments.

A visual representation of the entire experimental procedure, from adsorbent and adsorbate preparation to filtrate analysis, is presented in Fig. 1.

### Feedstock preparation

Walnut shells sourced from Italy were milled using a 2 mm grate in a Retsch ZM 200 Ultra Centrifugal Mill. A particle size of 1–2 mm was then obtained using a 1 mm sieve at an amplitude of 1.7 mm for 6 min in a Retsch AS 200 Vibratory Sieve Shaker. Shells were then dried for 48 h at 105 °C. Some shells then underwent further pre-pyrolysis treatment: treated shells were soaked at a concentration of 50 g*/*L in 200 mM NaOH for 68 h at room temperature, then washed with deionised (DI) water until neutral filtrate pH was achieved. Treated shells were then dried for 48 h at 105 °C.

Pretreatment of biomass with NaOH prior to pyrolysis is known to decrease the lignin content of biomass^66–69^, to decrease the degree of cellulose polymerisation^66,67^, , to remove acetyl and uronic acid substitutions from hemicellulose^68^, and to solubilise and remove some hemicellulose^66,69^ Furthermore, alkaline pretreatment is known to increase the porosity of biomass^67–69^.

In previous work, the authors have shown that (1) biomass pretreated with alkali will shrink at lower temperatures and gain less porosity upon pyrolysis than those not pretreated in this manner;^37^ and (2) that alkaline pretreatment of walnut shells increases pore surface hydrophilicity of derived biochars^70^.

### Pyrolysis

**Fig. 2:**
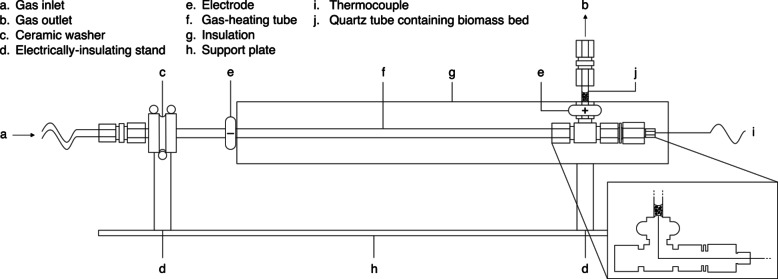
Schematic of the pyrolysis reactor used to produce biochars. Reprinted from Barr et al.^70^ with permission from Elsevier.

Biochars were produced using a pyrolysis reactor developed in house for in-situ imaging (see Fig. 2). 6 mm-tall beds of biomass were fixed between two stainless steel meshes in 3 mm inner diameter, 1.5 mm thick quartz tubes. Beds were convectively heated by a 3 L*/*min stream of resistively preheated argon at a rate of 6 °C*/*min to peak temperatures of 250, 350 and 450 °C. Beds were held at peak temperature for 30 min before cooling under the same gas flowrate. When the bed temperature reached 70 °C, the quartz tube was removed, and the bed compacted to 3 mm using a sterilised needle. This was not observed to cause particle fracturing. The tube was then sealed at both ends using Parafilm^®^ M (Bemis Company, Inc.), and stored in a desiccator until needed for filtration experiments.

### Reagent and media preparation

Sterile saline was made by dissolving 9 g*/*L NaCl in ultra-pure water (Milli-Q). The solution was autoclaved and stored at room temperature until needed. Prior to experiments, an appropriate aliquot was obtained and sterile-filtered through a 0.22 μm syringe filter.

The bacterial growth media used were tryptone soy broth (TSB) and tryptone soy agar (TSA). TSB powder was obtained from Thermo Fisher Scientific (Oxoid, product code CM0129); agar powder was purchased from Sigma Aldrich (CAS number 9002-18-0). Manufacturer instructions were observed in determining the following quantities: a concentration of 30 g*/*L of TSB in ultra-pure water (Milli-Q) was used for all bacterial growth in liquid TSB culture; the same concentration was used for solid TSA plates, with the addition of 15 g*/*L agar powder. All media were autoclaved within 1 h of manufacture.

### Bacterial strains

One Gram-positive and one Gram-negative organism were selected for this study, to determine whether the adsorption performance of the biochars was affected by bacterial surface chemistry. The Gram-positive organism was a clinical isolate of epidemic multidrug-resistant *Staphylococcus aureus* (EMRSA-15);^71^ the Gram-negative, a clinical strain of multidrug-resistant *Pseudomonas aeruginosa* (RP73) isolated from the lungs of a chronically-infected cystic fibrosis patient^72^. The EMRSA-15 isolate was acquired from the UK National Collection of Type Cultures (NCTC), with accession number NCTC 13,616. The RP-73 strain was generously donated by Dr Richard Amison, Institute of Pharmaceutical Science, King’s College London.

### Bacterial preparation

All microbiological work was carried out with sterile reagents and tools in a biological safety cabinet, following proper aseptic technique. Bacteria were streaked onto TSA plates from 30% glycerol stocks stored at -80 °C. These plates were incubated for 24 h at 37 °C and subsequently stored at 4 °C until needed for at most 2 weeks.

On the day prior to a filtration experiment, 10 mL of TSB in a 50 mL polypropylene centrifuge tube (Thermo Fisher Scientific, Greiner Bio-One Cellstar^®^ Catalogue Number 210 261) was inoculated with a single colony picked from the relevant plate. The liquid culture was incubated overnight (*≈* 20 h) at 37 °C, shaking at 180 rpm. A “blank” culture, consisting only of TSB, was grown alongside the two bacterial strains to ensure that growth was not due to media contamination.

On the day of the filtration experiment, the bacteria were spun down in a Labofuge 400R centrifuge at 4500 rpm (3939 *× g*) for 20 min. The supernatant was removed and the pellet resuspended in 10 mL sterile saline. A tenfold dilution was performed in the same sterile saline, and the optical density at 600 nm (OD_600_) was obtained using a Jenway 6300 spectrophotometer. The appropriate dilution was then made to adjust the OD_600_ to a known optical density (0.015 for RP73, 0.076 for EMRSA-15), such that the concentration of the final suspension was 10^7^ CFU/mL (colony forming units per millilitre). This concentration of bacteria was selected for both practical reasons (as the OD_600_ was easily monitored in the spectrophotometer with no dilution) and to represent heavily contaminated wastewater. Previous studies in India have shown concentrations of bacteria in wastewater up to 8.3* × *10^6^ CFU/mL^73^, which is the same order of magnitude as detected in studies from Slovakia^74^.

### Wastewater preparation

Raw wastewater influent was obtained from a major sewage treatment works in the UK, encompassing municipal and hospital sewage. Daily samples were collected over an eight-day period (22nd to 29th October 2021) and pooled to create a composite sample which was used for all subsequent wastewater experiments. A mix of 22 deuterated standards (purity *>* 97%, see Supplementary Tables 1–2) encompassing a wide range of physicochemical properties were added to the composite wastewater (final concentration of 500 ng*/*L).

### Filtration experiments

Quartz tubes containing samples (either biochars or raw feedstock) were placed into sterile 1.5 mL microcentrifuge tubes (Starlab TubeOne^®^, Catalogue Number S1615-5500). For all filtration experiments (bacterial and wastewater), the samples were “wetted” by loading 100 µL of sterile saline in the upper compartment of the tube, i.e. above the samples, and subsequently centrifuging them for 5 min at 1000 rpm (equivalent to 73 × *g*). This initial filtrate (< 100 µL) was discarded, and (in the case of bacterial filtration experiments) the procedure repeated. The subsequent filtrate (exactly 100 µL) was then recovered using a micropipette, and 50 µL were plated on TSA plates as a sterility control (i.e. to ensure that there was no existing contamination in the biochars prior to the experiment). Following this, an adequate volume of bacterial suspension (100 µL) or spiked wastewater (125 µL) was loaded and spun through in the same manner. This was repeated two to ten times (using a new sterile microcentrifuge tube each time).

### Filtrate analysis: bacteria

To investigate adsorption capacity, each of ten bacterial filtrates per biochar sample, as well as the unfiltered bacterial suspension, was serially diluted in sterile saline by factors of 10^4^, 10^5^, and 10^6^. 50 µL of each dilution was plated on TSA using approximately 25 sterile glass beads, and incubated at 37 °C overnight. After 24 h, colonies were counted, and CFU/mL concentrations of the undiluted filtrates were calculated.

Based on the viability counts from this experiment, a dilution factor of 10^5^ and a total of two filtrations per biochar sample were selected to reduce labour and material costs. A modified Miles and Misra technique was employed to determine viability count in subsequent experiments. The stock of bacterial suspension, made up to the OD_600_ specified above, was diluted by 10^5^ and three separate 10 µL drops were pipetted onto the surface of a TSA plate. An aliquot of the suspension was then filtered through the relevant sample, and three separate 10 µL drops of the filtrate placed onto a separate part of the same TSA plate. This process was repeated for the second filtration, with drops of the second filtrate placed onto yet another part of the same TSA plate. Each plate was incubated at 37 °C overnight. After 24 h, colonies were counted, and CFU/mL concentrations of the undiluted filtrates were calculated. Bacterial adsorption was subsequently calculated by dividing the CFU/mL concentrations of the filtrate by that of the original bacterial suspension.

### Filtrate analysis: wastewater

The organic solvents methanol (MeOH) and acetonitrile (MeCN) were purchased from Sigma Aldrich (Gillingham, Dorset, UK). LC-MS grade formic acid was obtained from Millipore (Bedford, USA). All chemical reagents used were of analytical grade or higher.

Filtrate was passed through a 0.2 μm PTFE nano filter vial (Thompson Instrument Company, Oceanside, CA, USA) before being subdivided into two aliquots of equal volume. To account for matrix effects, one aliquot was spiked (compounds added) to 500 ng*/*L using the same deuterated standards mix as above and the same volume of MeOH was added to the other to maintain the dilution factor between the two samples.

All samples were analysed using a rapid, highly sensitive LC-MS/MS method^65,75^ performed using a LCMS-8060 (Shimadzu Corporation, Kyoto, Japan) with a Raptor 5.0 × 3.0 mm, 2.7 μm biphenyl guard column (Restek, Pennsylvania, USA). Separations were performed using a binary gradient program consisting of an initial hold at 10% mobile phase B (MPB, 0.1% v/v formic acid in 50:50 MeOH: MeCN) for 0.2 min, followed by a linear ramp to 60% MPB over 2.8 min, and a hold at 100% MPB for 1 min, before returning to initial conditions for 1.5 min. The total method run time was 5.5 min, including the re-equilibration period, and mobile phase A consisted of 0.1% v/v formic acid (aq). The injection volume was 10 µL with a flow rate of 0.5 µL*/*min. Compounds were identified using multiple reaction monitoring (MRM) using at least two transitions (where possible) and a matching retention time to reference standards.

### Scanning electron microscopy

Samples were mounted on aluminium stubs using EM-Tec CT6 high purity conductive double sided adhesive carbon tabs (Labtech International Limited, Product No. 15–000406) and gold coated at a current of 20 mA under a 0.08 mbar vacuum for a minimum of 3 min using an Agar Scientific Automatic Sputter Coater in manual mode. Samples were then imaged in an FEI Inspect F scanning electron microscope using a field emission source with an accelerating voltage of 5 kV and an objective lens aperture of 30 μm. Images were acquired at working distances between 9 mm and 12 mm with a dwell time of 3 µs per pixel using four image integrations and the in-built “Drift Correction” function.

Scanning electron microscopy (SEM) was used to examine the micrometre- and nanometre-scale morphology of walnut shell and derived biochar surfaces and features (such as pit membranes and cell walls). In previous work^37^, the morphological evolution of these biochars during the pyrolysis process was characterised using X-ray computed tomography (XCT). This type of morphological analysis captures a key metric that more common analyses like Brunauer–Emmett–Teller (BET) gas adsorption analysis can not: surface accessibility. BET theory can be used to calculate a pore size distribution, but does not indicate where that porosity is located within a particle. Conversely, Barr et al.^37^. describe where porosity is located with respect to particle surfaces, finding that it tends to concentrate towards particle centres with increasing peak pyrolysis temperature.

### Statistics

All statistical analyses were performed in MATLAB, Microsoft Excel, and RStudio. Paired 2-tailed t-tests were performed to compare filtration numbers for each condition (*n* = 2). Independent two-sample equal variance t-tests were performed to compare each pair of temperatures within each combination of biomass treatment and bacterium (*n* = 4). Independent two-sample equal variance t-tests were also performed to compare untreated and pretreated biochars within each bacterium (*n* = 16) and overall (*n* = 32), and to compare between bacteria for all biochars (*n* = 32). One-way Analysis of Variance (ANOVA) with a Tukey post-hoc test was used to compare removal between biomass treatments (*n* = 16) and temperatures (*n* = 8) for each API and between APIs (*n* = 32). The same technique was used to compare removal of all APIs between treatments for each temperature (*n* = 88) and between temperatures for both treatments (*n* = 176). All significant differences between means identified are indicated in figures and/or supplementary information by the symbol *, signifying 0.005 ≤ *p* < 0.05, **, signifying 0.0005 ≤ *p* < 0.005, or ***, signifying *p* < 0.0005.

## Results and discussion

### Bacterial filtration

#### Adsorption capacity

**Fig. 3:**
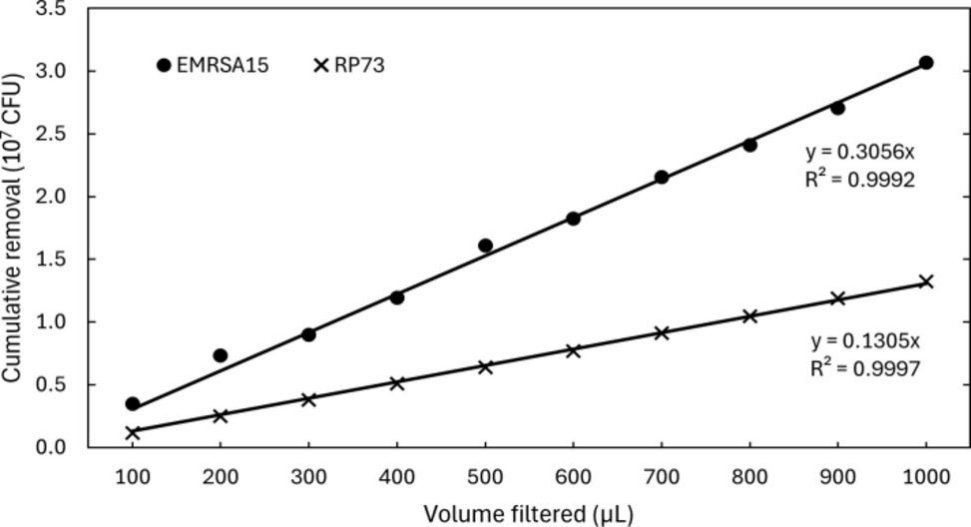
Cumulative removal of bacteria from a saline suspension (1.62 *×* 10^7^ CFU/mL RP73 or 5.44 *×* 10^7^ CFU/mL EMRSA-15) by filtration through a bed of pretreated walnut shell biochars pyrolysed at 450 °C by volume filtered. CFU: colony forming units.

To investigate the bacterial adsorption capacity of biochars, a serial filtration experiment was carried out (Fig. 3). Each bacterium was tested with one sample of alkali-pretreated biochar pyrolysed at a peak temperature of 450 °C. The difference in slope observed between the two bacteria (see Fig. 3) is primarily due to a slight difference in the concentration of the unfiltered suspension. As adsorption efficacy was not found to decrease over a period of 10 filtrations, tests comparing adsorption performance across production conditions were limited to two filtrations for practicality.

#### Effects of biochar production conditions

**Fig. 4:**
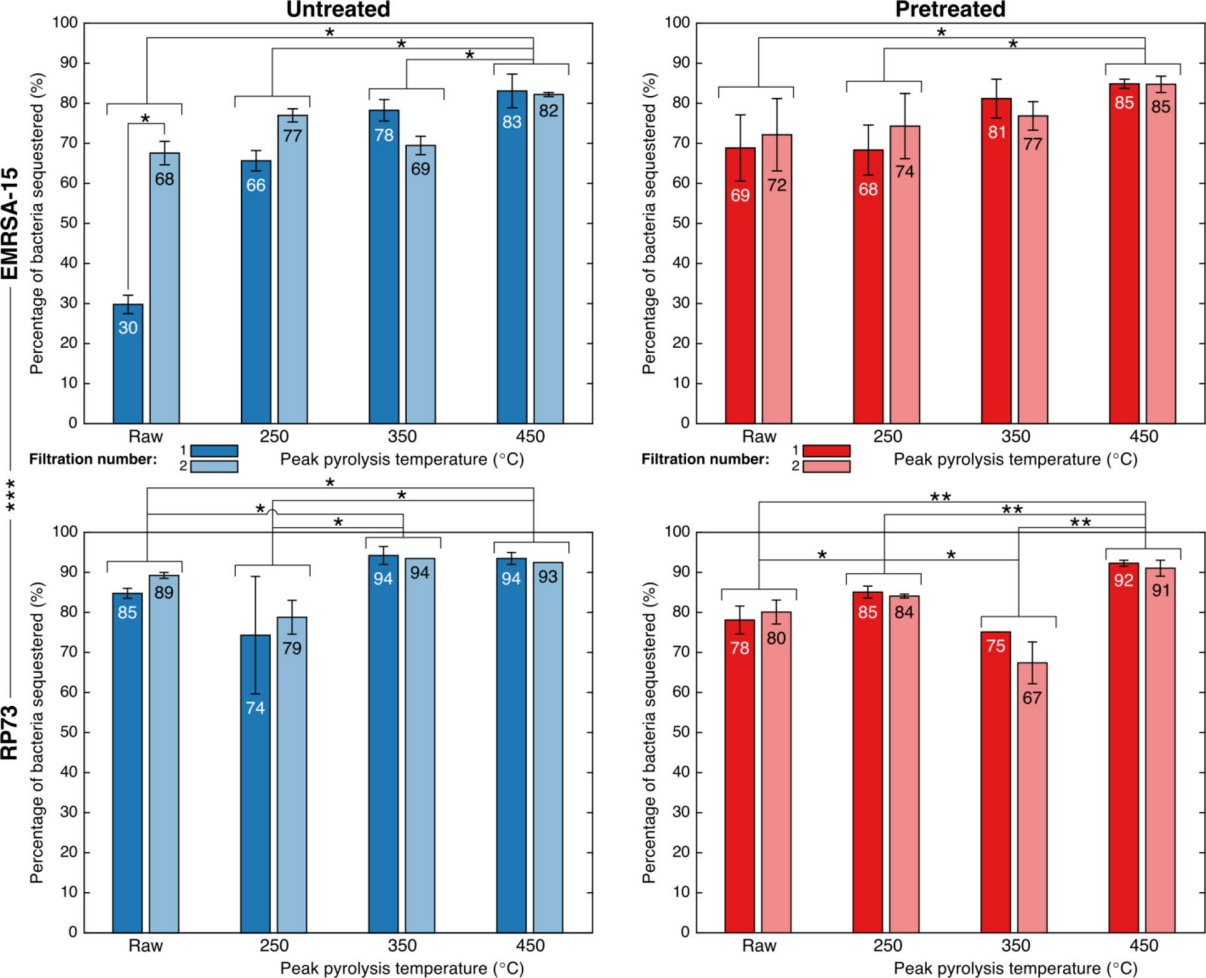
Average percentages of bacteria removed from a saline suspension by filtration through a bed of walnut shells or derived biochars by peak pyrolysis temperature, alkaline pretreatment, bacterial strain, and filtration number. Error bars represent the range of results between two independent batches of adsorbent produced under the same conditions. *: 0.005 ≤ *p* < 0.05; **: 0.0005 ≤ *p* < 0.005; ***: *p* < 0.0005.

On average, walnut shell biochars removed between 71% and 94% of saline-suspended bacteria over the course of two filtrations (Fig. 4). Removal efficacy was affected by peak pyrolysis temperature and alkaline pretreatment of biochars, as well as bacterial species filtered.

All biochars, except those pyrolysed at a peak temperature of 350 °C and pretreated with alkali solution, removed a greater proportion of the Gram-negative strain (*P. aeruginosa* RP73) than the Gram-positive strain (*S. aureus* EMRSA-15) tested. Untreated walnut shell biochars sequestered on average 88% of RP73, vs. 69% of EMRSA-15. Alkali-pretreated biochars sequestered on average 82% of RP73, vs. 76% of EMRSA-15. Across all biochar samples, the Gram-negative RP73 was sequestered more (*mean* = 85%; *SD* = 9%) than was the Gram-positive EMRSA-15 (*mean* = 73%; *SD* = 14%) in a statistically significant manner (*p* = 0.0001).

The burden of multidrug-resistant bacterial disease arises predominantly from Gram-negative bacteria, which are more difficult to treat in the clinic^3^, and represent a majority of both the “ESKAPE pathogens” (those species most often associated with antibiotic resistance), and the species on the WHO’s “Priority pathogens list for R&D of new antibiotics”^58,59^. The significantly superior adsorption of *P. aeruginosa* RP73 by the majority of tested biochars is therefore a beneficial feature.

No substantial deterioration in adsorption capacity was observed between the first and second filtrations performed, with certain samples adsorbing a greater proportion of bacteria during the second filtration than the first. For instance, untreated walnut shells pyrolysed at a lower peak temperature (250 °C) increased sequestration of EMRSA-15 from 66 to 77% and of RP73 from 74 to 79%. This suggests that maximum adsorption capacity was not the limiting factor with respect to adsorption performance, despite the high concentration of bacteria used in this laboratory-scale (rather than industrial-scale) experiment. Trends in adsorption performance differed according to the adsorbate being tested: sequestration of EMRSA-15 tended to increase with peak pyrolysis temperature, whereas RP73 was removed least effectively by biochars pyrolysed at a peak temperature of 250 °C when untreated, and 350 °C when pretreated with an alkaline solution (Fig. 4).

Overall, adsorption performance increased as peak pyrolysis temperature increased. Most of the statistically significant differences in adsorption efficacy were attributable to peak pyrolysis temperature. For EMRSA-15, there was a statistically significant difference in adsorption between untreated biochars with peak pyrolysis temperatures of 450 °C compared to all other temperatures (0.005 ≤ *p* < 0.05), and between pretreated biochars pyrolysed at 450 °C compared to those pyrolysed at 250 °C or left raw (0.005 ≤ *p* < 0.05). For RP73, there was a statistically significant difference in adsorption between untreated biochars pyrolysed at 450 °C compared to those pyrolysed at 250 °C or left raw (0.005 ≤ *p* < 0.05) and untreated biochars pyrolysed at 350 °C compared to those pyrolysed at 250 °C or left raw (0.005 ≤ *p* < 0.05). Pretreated biochars with peak pyrolysis temperatures of 450 °C also removed significantly more RP73 than those pyrolysed at all other temperatures (0.0005 ≤ *p* < 0.005). This may be attributed to the greater surface area of biochars produced at higher temperatures, resulting from production conditions that have been shown to increase porosity and, particularly as regards walnut shells pretreated with alkali, increase surface roughness^37,70^

A deviation from the overall trend of peak pyrolysis temperature being associated with higher adsorption efficacy can be seen when considering RP73. For this bacterium, raw, untreated walnut shells had higher adsorption efficacy compared to those pyrolysed at a peak temperature of 250 °C, though this difference was not statistically significant. Pretreated shells pyrolysed at a peak temperature of 250 °C had higher adsorption efficacies than those pyrolysed at a peak temperature of 350 °C (0.005 ≤ *p* < 0.05). This deviation may be attributed to decreasing biochar hydrophilicity. These ranges correspond directly to those over which the most substantial declines in biochar hydrophilicity were observed, as described in a previous publication^70^.

While decreased hydrophilicity sometimes outweighed increased surface area with respect to RP73 adsorption, this was not the case for EMRSA-15 adsorption. This is counter-intuitive as Gram-positive bacteria generally have a greater net surface charge than do Gram-negative bacteria^76^. This derives in part from the lipid membrane composition of these bacteria. Gram-positive bacterial membranes comprise only phosphatidylglycerol (PG), which is anionic at neutral pH. In contrast, Gram-negative bacteria have two membranes: an inner membrane rich in PG, and an outer membrane composed of a mixture of PG and phosphatidylethanolamine, which is zwitterionic at neutral pH. Further research leveraging multiple species of each type (Gram-negative and Gram-positive) with well-defined surface chemistries would help elucidate the specific physicochemical properties governing the interaction between the bacterium and the biochar. This would also help to differentiate between effects of bacterial morphology and surface chemistry.

In all cases, peak bacterial adsorption capacity was observed for the biochars pyrolysed at the highest peak temperature tested (450 °C). In the case of untreated walnut shells sequestering RP73, there was little difference in adsorption between this top temperature and 350 °C. At these higher temperatures (350 °C to 450 °C), pretreated biochars were able to remove more EMRSA-15 than were untreated biochars, while untreated biochars were able to remove more RP73 than were pretreated biochars, suggesting that a blend of untreated and pretreated biochars may be optimal for adsorption of a variety of bacteria. This may also be linked to the surface chemistry of bacteria and biochars, as pretreatment was found to markedly increase the interaction strength of walnut shell biochars with polar solvents in previous work. ^70^ Greater surface polarity could explain the stronger interaction of more charged Gram-positive bacteria with pretreated biochars, while more neutral Gram-negative bacteria favour less polar untreated biochars.

### Wastewater filtration

Two sequential filtrations of wastewater were performed to quantify API adsorption performance of biochars. In the raw influent wastewater, 27 native compounds were detected using LC-MS/MS analysis with log*P* values ranging from − 1.3 to 6.1. Of these, three compounds (clarithromycin, sulphapyridine, and trimethoprim) are antibiotics which drive the development of AMR^75,77–79^. The remaining native API composition consisted of anaesthetics, anticonvulsants, antidepressants, antihistamines, cardiovascular medication, diabetes medication, illicit drugs, metabolites, nervous system stimulants, and painkillers including prescription opioids. The concentration of these compounds was not determined in this work, but the average concentration of these compounds in London influent wastewater quantified in other studies were up to 2784 ± 148 ng/L (benzoylecgonine, the major urinary metabolite of the illicit drug cocaine)^65,80^. The percentage removal of non-deuterated (native) APIs was not quantified due to matrix effects.

**Fig. 5:**
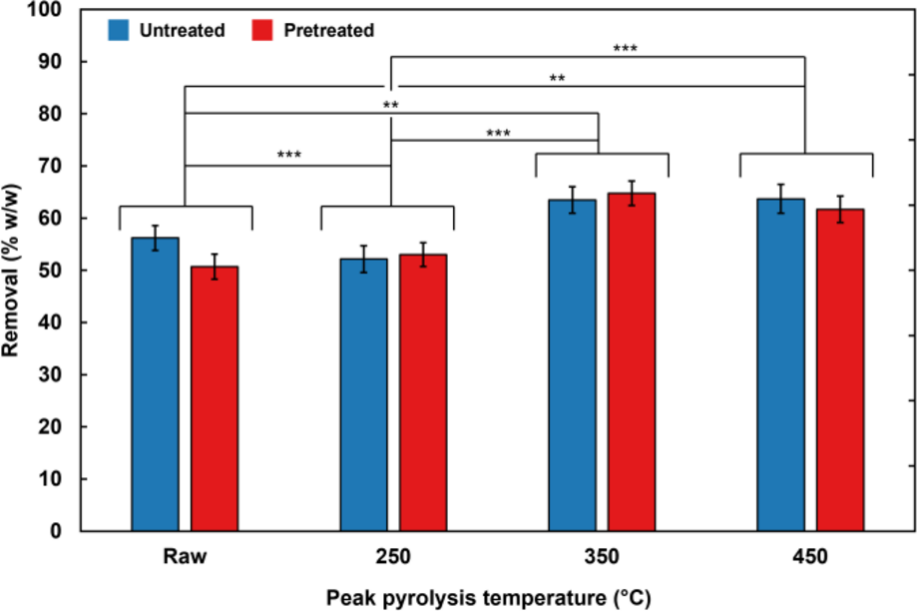
Average percentages of deuterated active pharmaceutical ingredients removed from wastewater by filtration through a bed of walnut shells or derived biochars by peak pyrolysis temperature and alkaline pretreatment. Error bars represent standard error (*n* = 88). *: 0.005 ≤ *p* < 0.05; **: 0.0005 ≤ *p* < 0.005; ***: *p* < 0.0005.

Selected compounds in their deuterated form (which does not occur naturally in the environment) were added to the influent wastewater before filtration for quality control purposes and used to quantify the removal of compounds by the biochar. On average, across all biochar production conditions, both filtration events, and all compounds, 58% ± 10% of deuterated APIs were removed from the treated effluent wastewater. Between the two filtration events across all biochar manufacturing conditions, there was no significant difference in the percentage removal across all compounds, 58% ± 13% and 58% ± 12% for the first filtration and second filtration, respectively. This suggests that the after two filtration events, the biochar had not become saturated and had the capacity to continue to remove deuterated APIs from wastewater. Similarly, there was no significant difference in deuterated API removal between pretreated and untreated biochars across all pyrolysis conditions (59% ± 10% and 58% ± 10%, *p* > 0.05). There were, however, significant differences in API removal driven by pyrolysis temperature (see Fig. 5).

The greatest removal of deuterated APIs was observed at peak temperatures of 450 °C and 350 °C (see Fig. 5). The average removal of all deuterated APIs by untreated biochars followed the same trend as that observed for removal of RP73 (minimised at 250 °C), while removal by pretreated biochars followed the same trend observed for EMRSA15 (broadly increasing with peak temperature). This suggests that API adsorption to untreated biochars was more affected by changes in biochar hydrophilicity than was that to pretreated biochars.

**Fig. 6:**
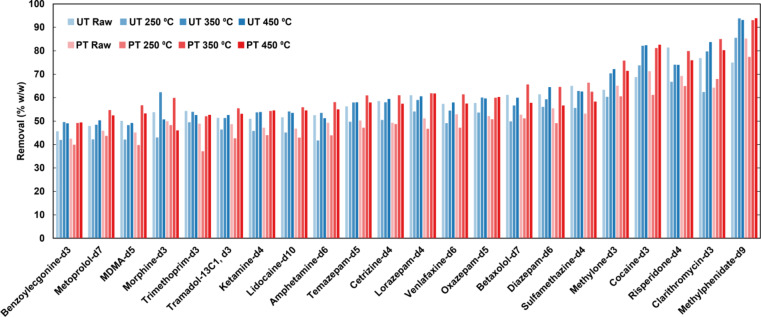
Average percentages of deuterated active pharmaceutical ingredients removed from wastewater by filtration through a bed of walnut shells or derived biochars by compound, alkaline pretreatment, and peak pyrolysis temperature. UT: untreated; PT: pretreated. Refer to Supplementary Tables 3 and Supplementary Fig. 1 for statistical significance.

Overall, the stimulant methylphenidate was highly removed by the biochars, with an average removal of 87% ± 10% across all filtrations, treatments, and peak pyrolysis temperatures. Biochars pyrolysed to a peak temperature ≥ 350 °C removed *>* 90% of methylphenidate, regardless of pre-treatment conditions (see Fig. 6). Other compounds that were well removed by the biochar (*>* 70% removal) include the macrolide antibiotic clarithromycin (75% ± 10%), the illicit stimulant cocaine (75% ± 10%), and the antipsychotic risperidone (73% ± 10%) (see Fig. 6).

**Fig. 7:**
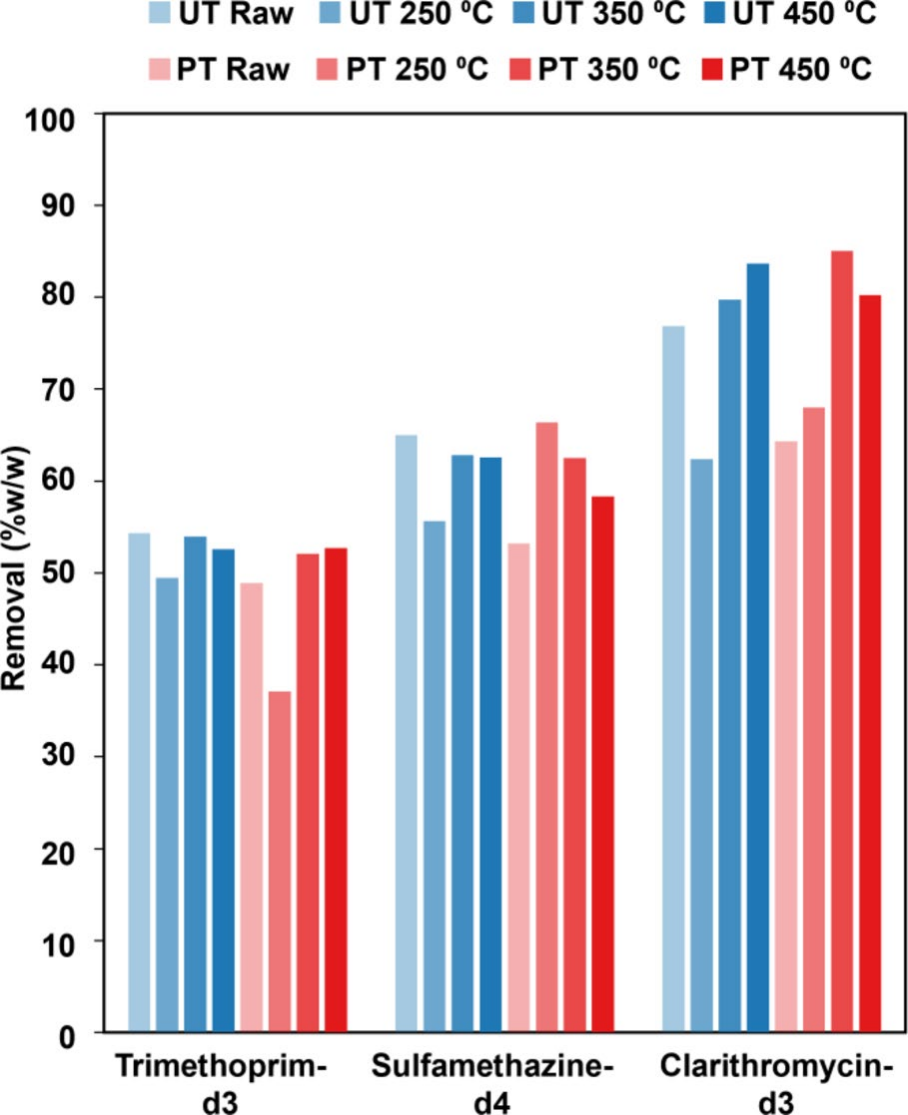
Average percentages of active pharmaceutical ingredients removed from wastewater by filtration through a bed of walnut shells or derived biochars by compound, alkaline pretreatment, and peak pyrolysis temperature for antibiotics. Refer to Supplementary Tables 3 and Supplementary Fig. 1 for statistical information.

Of particular interest to this work is the ability of biochars to remove antibiotics from wastewater to combat the spread of AMR in the environment. Of the three deuterated antibiotics tested (clarithromycin, sulfamethazine, and trimethoprim), biochars removed between 35% and 88% from WWTP influent (see Fig. 7). Untreated biochars performed slightly better than pretreated biochars at reducing concentrations of all antibiotics (63% ± 10% compared to 61% ± 10%), though this difference was not statistically significant. Removal of clarithromycin, sulfamethazine, and trimethoprim was maximised at 88%, 69%, and 55% using pretreated biochars pyrolysed at 350 °C, 250 °C and untreated biochars pyrolysed at 350 °C, respectively (see Supplementary Table 1).

Clarithromycin was the most effectively removed of the deuterated antibiotics measured. Of these three compounds, clarithromycin is the largest with a molecular weight of 750 Da compared to 290 Da and 278 Da for trimethoprim and sulfamethazine, respectively. Therefore, in addition to adsorption through chemical mechanisms such as hydrophobic interactions, hydrogen bonding, and other electron donor-acceptor interactions^81^, clarithromycin could be removed from the wastewater matrix by becoming trapped in the micropores of the biochar. Trimethoprim exhibited very low removal rates across all pre-treatment and pyrolysis conditions (50% ± 6%) when compared to other studies that reported a high affinity (*>* 80% removal) using other biochar feedstocks, including pistachio shells^82^, wood chips^83^, and rice husks^84^ across a range of pre-treatment and pyrolysis conditions. These differences could be partially attributed to the experimental design, where in previous studies the biochar was exposed to the antibiotic for at least 24 h before analysis while in this work the antibiotic was only briefly exposed to the biochar during centrifugation. Alternatively, the adsorption of trimethoprim onto the biochar substrate may have been inhibited by other constituents in the wastewater matrix that were not observed in previous work using an artificial aqueous solution. Further research is needed to determine what impact matrix effects from wastewater can have on the adsorption of APIs to biochar substrates.

In comparison to UK wastewater treatment plants employing activated sludge and trickle filter technologies as of 2019, biochars pyrolysed at 350 °C and 450 °C had greater removal rates for clarithromycin^85^. However, higher removal rates were observed at two urban wastewater treatment plants in the Republic of Ireland for all the antibiotic APIs than those observed in the biochar^86^. It is important to note that the wastewater treatment process contains multiple steps, unlike the simple filtration tested here, and the efficiency of API removal using current wastewater treatment technologies is highly variable depending on the type of treatment deployed^87^. On the whole, walnut shell biochars compare well to existing sludge-based technologies^86^, while providing an alternative waste valorisation pathway for LCB. These results demonstrate that biochar-based filters, designed with rationally selected production conditions, could be effectively employed in sequestering not only MDR organisms but also the antimicrobial pharmapollutants which most directly drive their selection in the environment. Recent studies have demonstrated the co-selective effects of all pharmapollutants on AMR, as opposed to just antimicrobials^88^. Thus, the ability of walnut shell biochars to reduce the overall concentration of pharmapollutants–removing no less than 50% of all compounds across all conditions–may reduce development of AMR in the environment, and thereby drug-resistant infections by MDR organisms.

### Biochar morphology

#### Surface accessibility

Scanning electron microscopy revealed that the surface accessibility of alkali-pretreated and untreated walnut shell biochars evolved during pyrolysis *via* rupture of pit membranes, expansion of extracellular space, and separation & fusion of cell wall layers.

The sclereid cells that compose walnut shells are connected by pits running from the lumen through secondary cell walls to a finely perforated pit membrane in the primary cell wall. These perforations average around 5 nm in diameter in homogeneous angiosperm pit membranes, with maximum diameters typically less than 100 nm^89^. Pit membranes are often paired across cells such that matter can move directly from one cell lumen to another. This mode of transport in plant tissue is called symplastic transport.

**Fig. 8:**
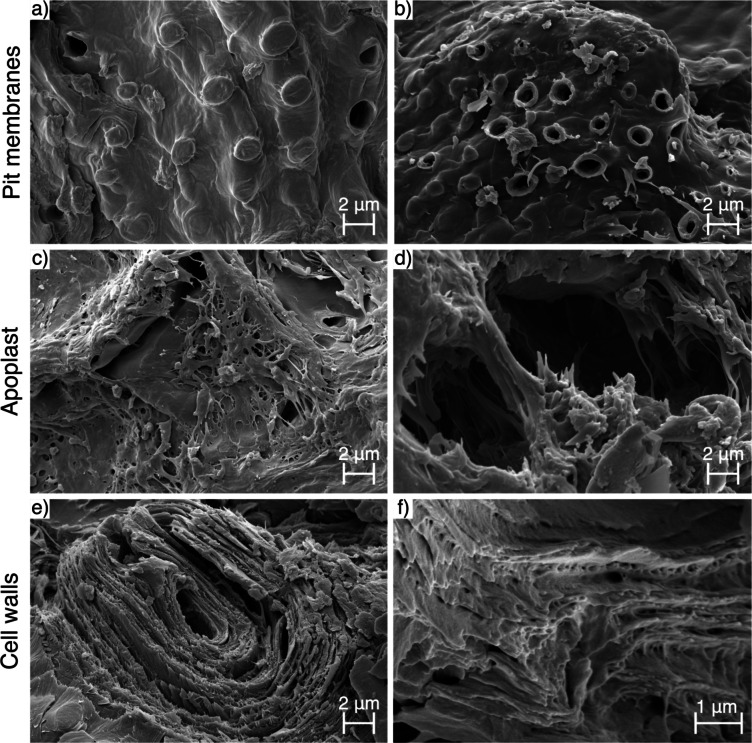
Scanning electron micrographs of (a-b) pit membranes in (a) raw pretreated walnut shells and (b) untreated walnut shell biochars pyrolysed at 450 °C; (c-d) the apoplast in untreated walnut shell biochars pyrolysed at (c) 350 °C and (d) 450 °C; and (e-f) cell walls in (e) raw untreated walnut shells and (f) pretreated walnut shell biochars pyrolysed at 250 °C.

Micrographs of walnut shells and derived biochars (Fig. 8a-b and Supplementary Fig. 2) show that pit membranes ruptured as a result of pyrolysis. This may be explained by a build-up of internal pressure caused by volatilisation of organic compounds inside cells during pyrolysis. While small molecules can theoretically pass through porous pit membranes, larger adsorbates (like bacteria) cannot access internal cell surfaces this way. However, in the absence of pit membranes, the pit canals connecting adjacent sclereid cells share approximately the same length scale as the bacteria considered (0.5 μm to 3 μm).

However, micrographs (Fig. 8b and Supplementary Fig. 2h) also show that some pit membranes do persist at peak temperatures as high as 450 °C. Moreover, micrographs of raw nutshells (Fig. 8a and Supplementary Fig. 2a) show that pits on the exposed surfaces of samples may sometimes be ruptured prior to pyrolysis by the mechanical stress of milling alone. Nonetheless, the rupture of pit membranes as pyrolysis proceeds offers an explanation for the observed increasing trends in bacterial adsorption efficacy with peak pyrolysis temperature.

The other main mode of transport in plant tissue is *via* the extracellular space, known as apoplastic transport. This relies on the porous middle lamella connecting adjacent cells as well as empty air gaps within nutshells (the apoplast). Micrographs (Fig. 8c and Supplementary Fig. 3) show that both smooth air gaps and web-like middle lamella structures persist even at the highest peak temperatures tested. In pretreated samples, finer strands in these structures are less prevalent (compare Supplementary Fig. 2b and f) and some fusion of the structure appears to occur as a result of the pretreatment (see Supplementary Fig. 3e).

At higher peak pyrolysis temperatures, there is some evidence of cells pulling apart, widening the apoplast. Figure 8d shows thin strands, thinnest at their centres and anchored to a cell at either end, as if they had been stretched as the cells pulled apart. This behaviour would increase extracellular porosity as well as the surface area accessible to large adsorbates. The secondary cell walls themselves also have some very fine porosity which may be accessible to small molecule adsorbates. The cell walls are composed of cellulose microfibrils in an incrementing helicoidal arrangement, which can be seen quite clearly in Fig. 8e. Here, the finely porous mass of lignin and hemicellulose between these layers of microfibrils may also be observed.

As discussed in a previous publication^70^, when walnut shells are pretreated with an alkaline solution, this mass between layers appears to recede (compare Supplementary Fig. 4a and e). Around 250 °C, biochars show more individual pores between layers (see Fig. 8f), rather than either a fine porous mass or empty space. This could be due to lignin beginning to volatilise below this temperature while cellulose is still relatively stable^90^. At peak temperatures *≥* 350 °C, cell wall layers appear to fuse (see Supplementary Fig. 4c-d and g-h). For this reason, peak pyrolysis temperatures around 250 °C may represent a maximum in surface area accessible to small molecule adsorbates within cell walls. Small pores, like those present between cell wall layers, increase the static diffusion time of small molecules, thereby increasing adsorption efficiency by slowing efflux.

#### Surface texture

Surface texture affects the surface area to volume ratio of particles, and thus their adsorption capacity, as well as fluid mechanics in and around particles. The complex evolution of surface texture during pyrolysis of walnut shells was found to depend on pretreatment, position of the surface within the biological tissue, and native surface texture.

External surfaces of sclereid cells comprise the primary cell wall, which, like secondary cell walls, is supported by a scaffold of cellulose microfibrils (causing it to appear striated), but contains more pectin and less lignin than do secondary walls. The native striation of the primary cell wall tended to appear smoother after alkaline pretreatment (compare Supplementary Fig. 5a and e) and as pyrolysis proceeds (see Supplementary Fig. 5a-f).

**Fig. 9:**
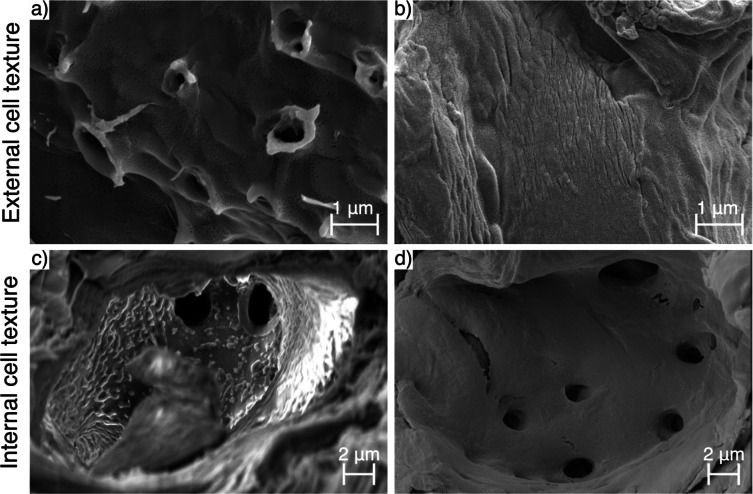
Scanning electron micrographs of (a-b) external cell texture in (a) untreated walnut shell biochars pyrolysed at 450 °C and (b) pretreated walnut shell biochars pyrolysed at 350 °C; and (c-d) internal cell texture in (c) untreated walnut shell biochars pyrolysed at 250 °C and (d) raw pretreated walnut shells.

As discussed in a previous publication^70^, untreated walnut shells develop mild pitting on external cell surfaces at a peak temperature of 450 °C, particularly in the vicinity of what appear to be popped pit membranes (see Fig. 9a and Supplementary Fig. 5d). This implies that this pitting is the result of the release of reactive gases formed within cells during pyrolysis *via* the symplastic transport route.

Conversely, in pretreated biochars, a velvety texture was seen to develop by 350 °C (see Fig. 9b and Supplementary Fig. 5g), which further develops into a complex texture (wherein smaller deposits coat larger deposits), by 450 °C (see Supplementary Fig. 5h). It is important to note that this severe surface texture was not universal in these particles; some surfaces remained far smoother, or simply velvety in texture.

The internal surface of sclereid cells comprises the innermost secondary cell wall, sometimes thickened (depending on maturity) by a deposit of lignin and hemicellulose, as well as any remnants of the contents of the cytoplasm. This means that internal cell texture in raw walnut shells varies greatly. Where present, a rough internal texture appears able to withstand pyrolysis (see Fig. 9c and Supplementary Fig. 6c), but not alkaline pretreatment (see Fig. 9d). In cells with a smoother internal texture (either natively or due to pretreatment), this tends to evolve similarly to external surface texture (see Supplementary Fig. 6d-h).

The surface texture observed is too fine to increase the surface area available to large adsorbates like bacteria, but could contribute to increasing adsorption capacity for small molecules like APIs. This suggests that API adsorption performance would be maximised under high temperature and alkaline pretreatment conditions. While peak temperature did have the expected effect, the same was not observed for alkaline pretreatment, suggesting that the increase in surface area driving improved adsorption performance (of both APIs and bacteria) may be driven principally by increased surface accessibility.

## Conclusions

Waste lignocellulosic biochars showed excellent adsorption efficacy for both APIs that exert selective pressures and the MDR bacteria that are subject to these pressures. Walnut shell biochars removed up to 97% of MDR clinical bacterial isolates from a saline suspension. Adsorption performance was generally maintained during a second filtration of fresh suspension, and the best performance achieved at the highest peak pyrolysis temperature tested (450 °C). For the most part, walnut shells and derived biochars were able to remove a greater percentage of *P. aeruginosa* RP73 than *S. aureus* EMRSA-15. Moreover, at peak pyrolysis temperatures *≥* 350 °C, alkaline pretreated biochars were able to remove more of the Gram-positive bacterial strain (*S. aureus* EMRSA-15) than were untreated biochars and vice versa. Biochars produced at higher peak pyrolysis temperatures (350 °C and 450 °C) were most effective at removing APIs from wastewater, including up to 97% of methylphenidate, and 88% of the antibiotic clarithromycin. This shows that production conditions alone can modulate the ability of biochar to sequester bacteria in a species-dependent manner, and APIs as a function of physicochemical properties. These results will inform the rational design of blended biochar wastewater filters for sequestration of diverse pollutant adsorbates.

Suggestions of the morphological mechanisms underlying these findings are provided by SEM. Micrographs showed that internal surfaces in biochars became more accessible as pyrolysis proceeded due to the popping of pit membranes between cells and the spreading of cells apart from one another. However, area between cell wall layers was most accessible at low pyrolysis temperatures (250 °C). Surface texture was also found to roughen considerably as pyrolysis proceeded, most drastically in the case of pretreated shells pyrolysed at 450 °C. As pretreatment was not found to significantly increase the average API adsorption efficacy of biochars, surface accessibility is likely the primary morphological factor driving improved adsorption performance with increasing peak pyrolysis temperature. While APIs could theoretically fit through perforations in pit membranes and in pores between cell wall layers, bulk flow of the aqueous matrix is likely to take a less restricted path. Therefore, the popping of pit membranes and spreading of cells as pyrolysis proceeds are the most likely factors to be driving a substantial increase in the surface area available for adsorption of both bacteria and APIs as pyrolysis proceeds.

Having shown successful sequestration of pollutants that drive the spread of environmental AMR, future work must consider the life cycle of biochar wastewater filters. Being derived from a resource that would otherwise have been a waste stream, they are undoubtedly sustainable, but to determine whether they are also circular, sterilisation methods such as re-pyrolysis to enable reuse (or at least safe disposal) need to be tested. Nonetheless, these results represent a promising development towards waste valorisation for pollution prevention in the context of AMR mitigation and One Health. They should be considered a jumping off point for further exploration of effects of biochar production conditions on in-line wastewater filtration performance.

## Electronic supplementary material

Below is the link to the electronic supplementary material.

Supplementary Material 1

## Data Availability

The authors declare that all processed data supporting the findings of this study are available within the paper and its Supplementary Information files. Additional raw data files are available from the corresponding author upon reasonable request.
