# Supplementary material for: Biochar filtration of drug-resistant bacteria and active pharmaceutical ingredients to combat antimicrobial resistance
Source: Sci Rep. 2025 Jan 8;15:1256. doi: 10.1038/s41598-024-83825-2 (PMC11711200; doi:10.1038/s41598-024-83825-2)
Supplement: Supplementary file 1 — Supplementary Material 1 [file 41598_2024_83825_MOESM1_ESM.pdf]

Biochar filtration of drug-resistant bacteria and active pharmaceutical ingredients to combat antimicrobial resistance

Paul-Enguerrand Fady, Alexandra K. Richardson, Leon P. Barron,
A. James Mason, Roberto Volpe, and Meredith R. Barr

Supplementary Table 1. Average percent removal (standard error) of each API using untreated walnut shells and derived biochars by peak pyrolysis temperature and filtration event. Log*P* data sourced from Chemspider and PubChem

Compound	Log*P*	RAW-1	RAW-2	250-1	250-2	350-1	350-2	450-1	450-2
Benzoylecgonine-d3	-0.2	45 (1)	46 (2)	40 (1)	44 (2)	49 (2)	50 (1)	48 (0.03)	50 (0.08)
Amphetamine-d6	-0.6	49 (1)	56 (0.01)	44 (2)	40 (1)	56 (0.3)	51 (4)	52 (3)	50 (2)
Sulfamethazine-d4	0.3	68 (0.7)	62 (0.7)	58 (2)	53 (7)	66 (1)	59 (3)	65 (5)	60 (6)
Methylone-d3	1.0	61 (3)	66 (1)	60 (3)	60 (4)	72 (0.7)	68 (0.5)	71 (3)	73 (7)
Trimethoprim-d3	1	54 (2)	54 (0.2)	50 (1)	49 (1)	53 (3)	55 (0.7)	52 (2)	53 (2)
MDMA-d5	-0.8	49 (3)	51 (1)	43 (3)	41 (1)	49 (4)	47 (3)	49 (4)	49 (1)
Metoprolol-d7	-0.3	49 (0.02)	47 (2)	38 (2)	47 (2)	46 (2)	51 (2)	50 (2)	51 (0.4)
Tramadol-d3	0.5	50 (3)	53 (0.6)	45 (0.08)	48 (3)	53 (1)	50 (0.8)	53 (1)	52 (2)
Methylphenidate-d9	0.3	71 (2)	79 (1)	84 (3)	87 (3)	93 (0.1)	94 (0.2)	92 (0.1)	94 (0.5)
Oxazepam-d5	2.0	58 (0.5)	57 (2)	53 (3)	54 (0.7)	62 (1)	58 (2)	58 (0.9)	61 (0.3)
Lidocaine-d10	2.0	52 (0.5)	52 (0.03)	44 (0.07)	47 (3)	55 (2)	54 (2)	53 (0.07)	54 (0.9)
Lorazepam-d4	3.0	59 (3)	63 (2)	51 (4)	57 (2)	59 (3)	59 (3)	61 (0.8)	60 (2)
Ketamine-d4	2.0	51 (1)	51 (0.5)	45 (0.4)	47 (3)	53 (2)	54 (0.4)	54 (0.9)	53 (1)
Cetrizine-d4	3.0	59 (1)	59 (0.2)	49 (0.2)	52 (1)	58 (0.2)	58 (1)	60 (2)	59 (0.3)
Cocaine-d3	1.0	66 (2)	72 (2)	73 (2)	74 (4)	82 (0.5)	83 (0.5)	82 (0.4)	83 (0.8)
Venlafaxine-d6	1.0	56 (0.7)	59 (1)	48 (3)	50 (2)	58 (2)	51 (2)	60 (3)	56 (2)
Temazepam-d5	2.0	56 (0.3)	56 (0.06)	49 (0.04)	50 (2)	59 (2)	57 (0.5)	58 (2)	58 (1)
Betaxolol-d7	0.8	58 (3)	64 (1)	50 (1)	49 (1)	60 (2)	54 (0.8)	63 (5)	57 (4)
Risperidone-d4	2.0	83 (0.2)	80 (0.4)	68 (0.5)	65 (2)	80 (0.09)	68 (0.2)	78 (4)	70 (4)
Diazepam-d6	3.0	61 (1)	61 (0.2)	54 (2)	58 (5)	61 (7)	58 (1)	64 (0.5)	65 (3)
Morphine-d3	0.8	46 (14)	62 (1)	41 (1)	45 (0.5)	68 (0.02)	57 (7)	55 (0.7)	47 (17)
Clarithromycin-d3	2.0	76 (2)	78 (2)	65 (9)	60 (6)	84 (1)	76 (2)	87 (3)	80 (1)

Supplementary Table 2. Average percent removal (standard error) of each API using pre-treated walnut shells and derived biochars by peak pyrolysis temperature and filtration event. Log*P* data sourced from Chemspider and PubChem

Compound	Log*P*	RAW-1	RAW-2	250-1	250-2	350-1	350-2	450-1	450-2
Benzoylecgonine-d3	-0.2	47 (4)	38 (4)	39 (0.8)	41 (4)	49	49 (1)	48 (4)	51 (0.5)
Amphetamine-d6	-0.6	54 (5)	45 (8)	42 (8)	46 (0.5)	55	60 (4)	52 (6)	58 (0.7)
Sulfamethazine-d4	0.3	59 (4)	48 (1)	64 (4)	69 (3)	55	66 (4)	60 (7)	57 (7)
Methylone-d3	1.0	68 (5)	63 (0.4)	54 (2)	67 (0.6)	78	75 (0.2)	73 (10)	70 (2)
Trimethoprim-d3	1	52 (4)	46 (2)	35 (5)	39 (4)	56	50 (1)	52 (5)	54 (0.7)
MDMA-d5	-0.8	47 (10)	43 (5)	37 (4)	42 (3)	52	59 (2)	53 (7)	54 (2)
Metoprolol-d7	-0.3	51 (2)	41 (0.7)	39 (0.4)	48 (4)	49	57 (2)	52 (5)	53 (0.4)
Tramadol-d3	0.5	53 (6)	44 (2)	40 (0.9)	45 (2)	57	55 (3)	52 (4)	54 (1)
Methylphenidate-d9	0.3	86 (3)	85 (1)	78 (4)	77 (3)	97	91 (2)	93 (0.5)	94 (0.8)
Oxazepam-d5	2.0	57 (3)	47 (5)	48 (3)	54 (5)	55	63 (7)	59 (3)	61 (3)
Lidocaine-d10	2.0	52 (5)	42 (5)	41 (3)	45 (4)	55	56 (2)	53 (5)	56 (1)
Lorazepam-d4	3.0	54 (2)	48 (2)	47 (1)	46 (2)	60	63 (4)	63 (8)	61 (2)
Ketamine-d4	2.0	50 (6)	44 (3)	44 (0.03)	44 (4)	57	53 (0.8)	54 (4)	55 (2)
Cetrizine-d4	3.0	54 (5)	45 (4)	47 (0.5)	51 (4)	58	63 (3)	55 (5)	60 (0.9)
Cocaine-d3	1.0	73 (7)	70 (3)	59 (1)	63 (6)	89	77 (4)	82 (3)	83 (0.6)
Venlafaxine-d6	1.0	57 (7)	49 (1)	45 (0.9)	49 (2)	59	63 (3)	56 (4)	59 (3)
Temazepam-d5	2.0	54 (4)	46 (3)	45 (1)	49 (4)	59	62 (3)	56 (5)	60 (0.8)
Betaxolol-d7	0.8	56 (7)	50 (0.5)	51 (6)	51 (2)	62	68 (0.5)	56 (4)	59 (5)
Risperidone-d4	2.0	73 (10)	66 (0.08)	64 (0.8)	66 (3)	76	82 (1)	76 (4)	76 (7)
Diazepam-d6	3.0	60 (8)	51 (0.1)	47 (4)	51 (2)	50	72 (1)	53 (4)	60 (8)
Morphine-d3	0.8	52 (3)	48 (5)	43 (6)	53 (4)	63	58 (14)	51 (10)	41 (2)
Clarithromycin-d3	2.0	69 (8)	60 (3)	66 (7)	70 (5)	88	83 (0.4)	81 (0.7)	80 (8)

Supporting Information: Fady *et al*. Biochar filtration of drug-resistant bacteria and active pharmaceutical ingredients to combat antimicrobial resistance.

Supplementary Table 3. Statistical significance between the different APIs across treatments and temperatures.

	Amphetamine-d6																				
Benzoylecgonine-d3	-	Benzoylecgonine-d3																			
Betaxolol-d7	-	***	Betaxolol-d7																		
Cetrizine-d4	-	***	-	Cetrizine-d4																	
Clarithromycin-d3	***	***	***	***	Clarithromycin-d3																
Cocaine-d3	***	***	***	***	-	Cocaine-d3															
Diazepam-d6	*	***	-	-	***	***	Diazepam-d6														
Ketamine-d4	-	-	-	-	***	***	**	Ketamine-d4													
Lidocaine-d10	-	-	-	-	***	***	**	-	Lidocaine-d10												
Lorazepam-d4	*	***	-	-	***	***	-	*	*	Lorazepam-d4											
MDMA-d5	-	-	***	*	***	***	***	-	-	***	MDMA-d5										
Methylone-d3	***	***	***	***	**	**	***	***	***	***	***	Methylone-d3									
Methylphenidate-d9	***	***	***	***	***	***	***	***	***	***	***	***	Methylphenidate-d9								
Metoprolol-d7	-	-	***	*	***	***	***	-	-	***	-	***	***	Metoprolol-d7							
Morphine-d3	-	***	-	-	***	***	*	-	-	-	-	***	***	-	Morphine-d3						
Oxazepam-d5	-	***	-	-	***	***	-	-	-	-	***	***	***	***	-	Oxazepam-d5					
Risperidone-d4	***	***	***	***	-	-	***	***	***	-	***	-	***	***	***	***	Risperidone-d4				
Sulfamethazine-d4	**	***	-	-	***	***	-	***	**	-	***	-	***	***	***	-	***	Sulfamethazine-d4			
Temazepam-d5	-	***	-	-	***	***	-	-	-	-	*	***	***	*	-	-	***	-	Temazepam-d5		
Tramadol-d3	-	-	*	-	***	***	**	-	-	*	-	***	***	-	-	*	***	***	-	Tramadol-d3	
Trimethoprim-d3	-	-	*	-	***	***	**	-	-	*	-	***	***	-	-	*	***	***	-	-	Trimethoprim-d3
Venlafaxine-d6	-	***	-	-	***	***	-	-	-	-	*	***	***	*	-	-	***	-	-	-	-

*: $0.005 \le p < 0.05$;
**: $0.0005 \le p < 0.005$;
***: $p < 0.0005$

Supporting Information: Fady *et al*. Biochar filtration of drug-resistant bacteria and active pharmaceutical ingredients to combat antimicrobial resistance.

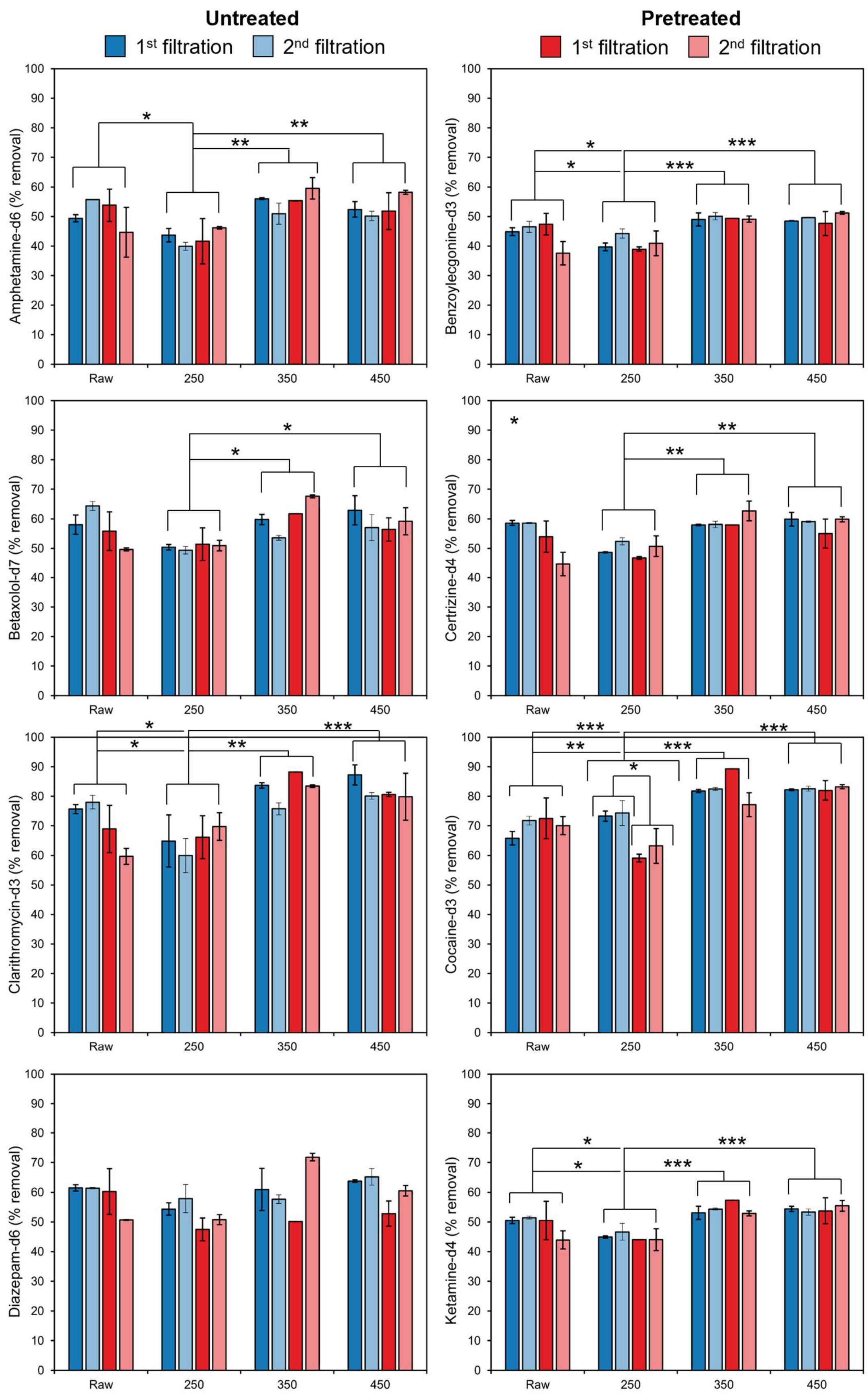

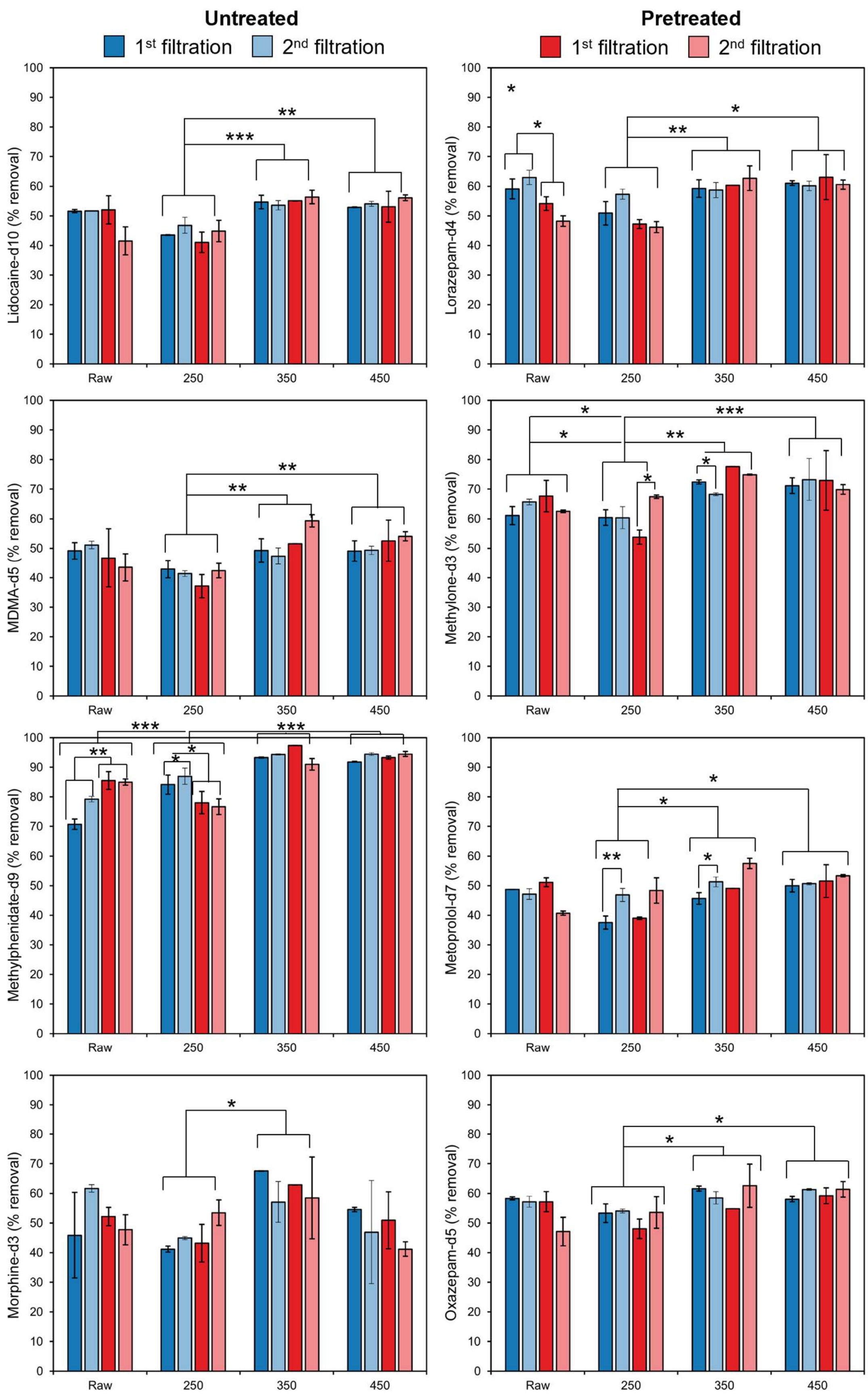
Untreated
1st filtration
2nd filtration
Pretreated
1st filtration
2nd filtration
Lidocaine-d10 (% removal)
Lorazepam-d4 (% removal)
MDMA-d5 (% removal)
Methylone-d3 (% removal)
Methylphenidate-d9 (% removal)
Metoprolol-d7 (% removal)
Morphine-d3 (% removal)
Oxazepam-d5 (% removal)
Raw
250
350
450

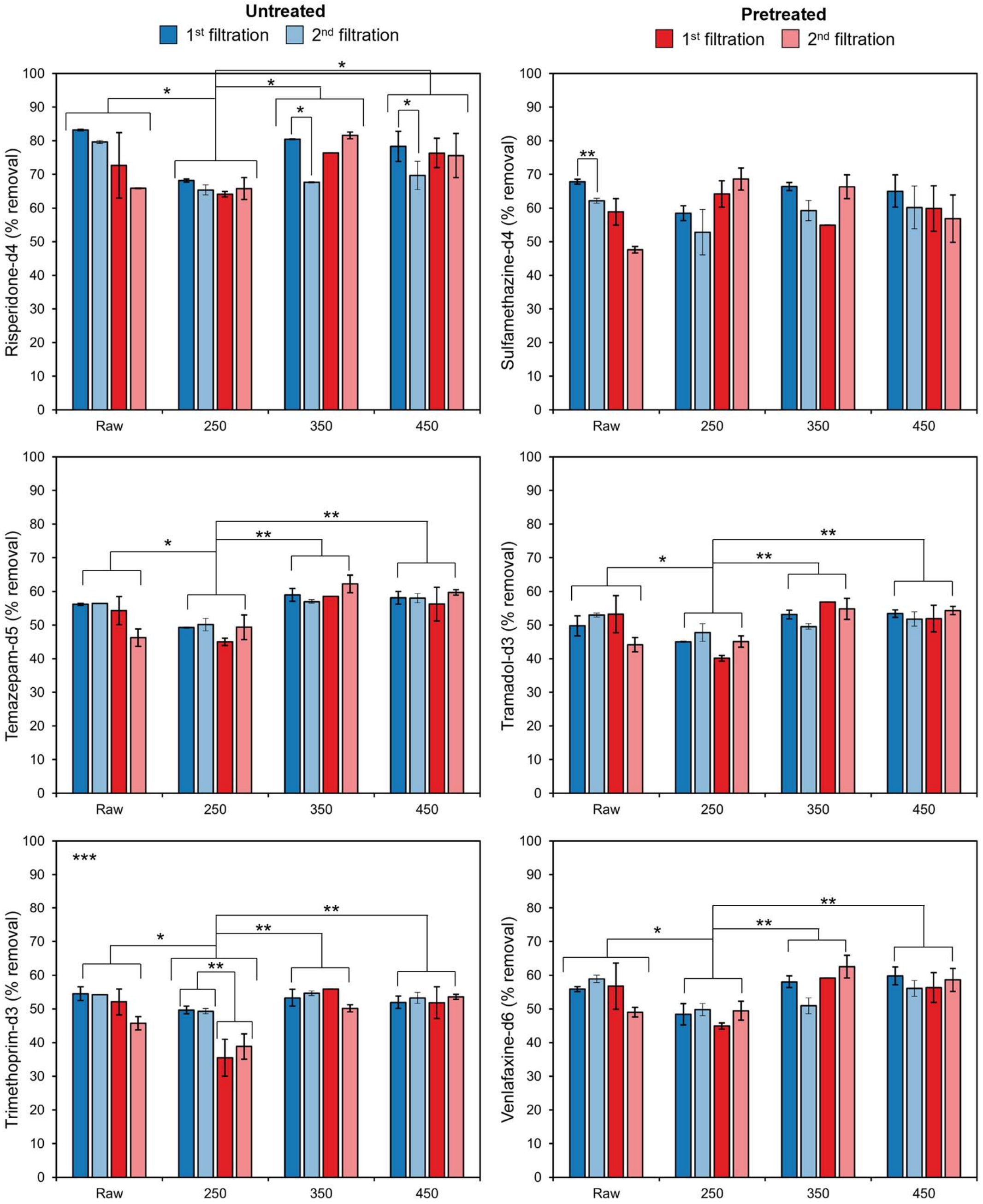

Supplementary Figure 1. Average percentages of deuterated active pharmaceutical ingredients removed from wastewater by filtration through a bed of walnut shells or derived biochars by peak pyrolysis temperature and alkaline pretreatment. Error bars represent the range of results between two independent batches of adsorbent produced under the same conditions. Asterix presence on the top left corner of each graph represents a significant difference between alkaline pretreated and untreated samples across all pyrolysis temperatures. *: $0.005 \leq p < 0.05$; **: $0.0005 \leq p < 0.005$; ***: $p < 0.0005$.

Supporting Information: Fady *et al*. Biochar filtration of drug-resistant bacteria and active pharmaceutical ingredients to combat antimicrobial resistance.

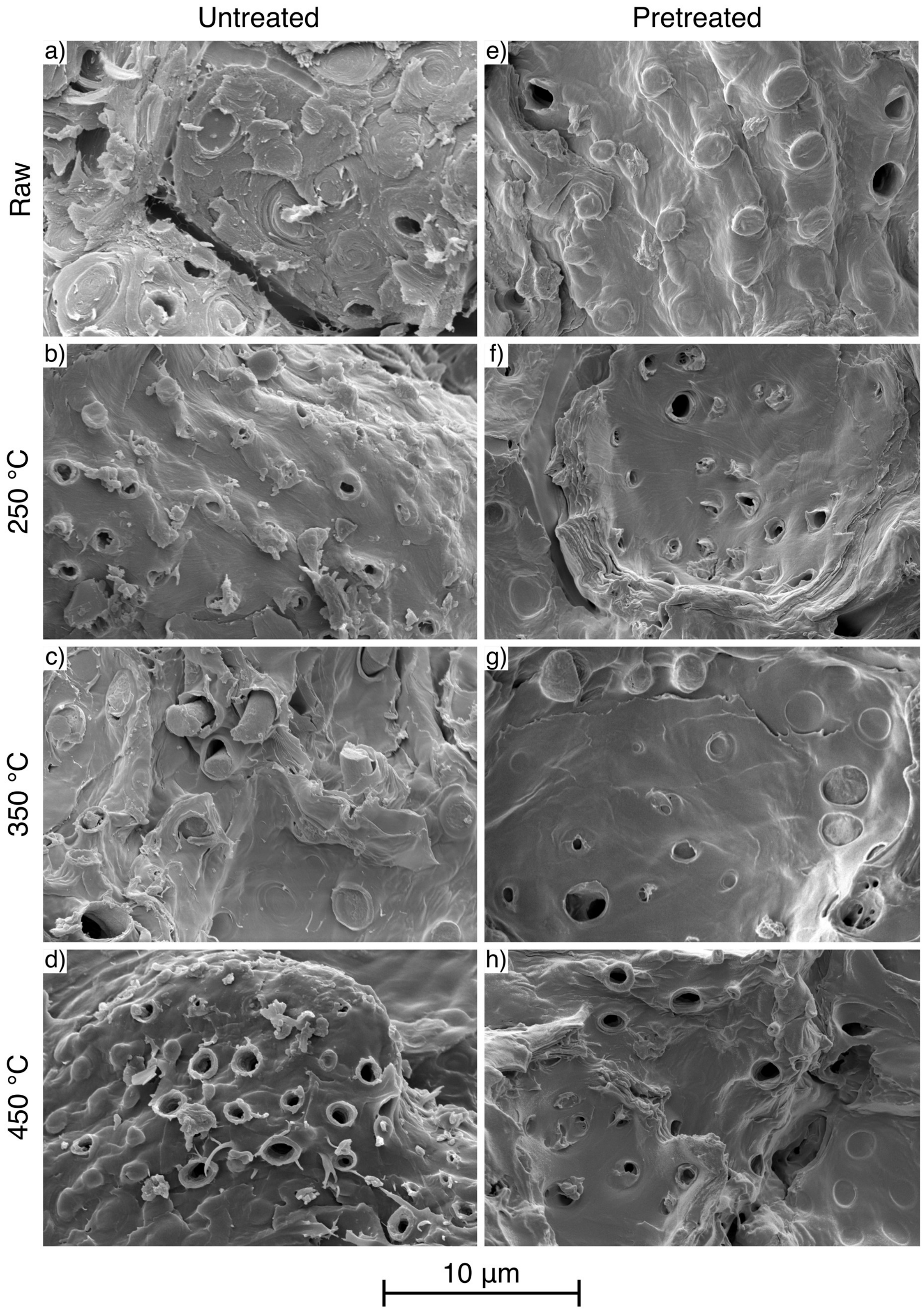

Supplementary Figure 2. Scanning electron micrographs of pit membranes in untreated (a-d) and alkali-pretreated (e-h) walnut shell particles and derived biochars by peak pyrolysis temperature.

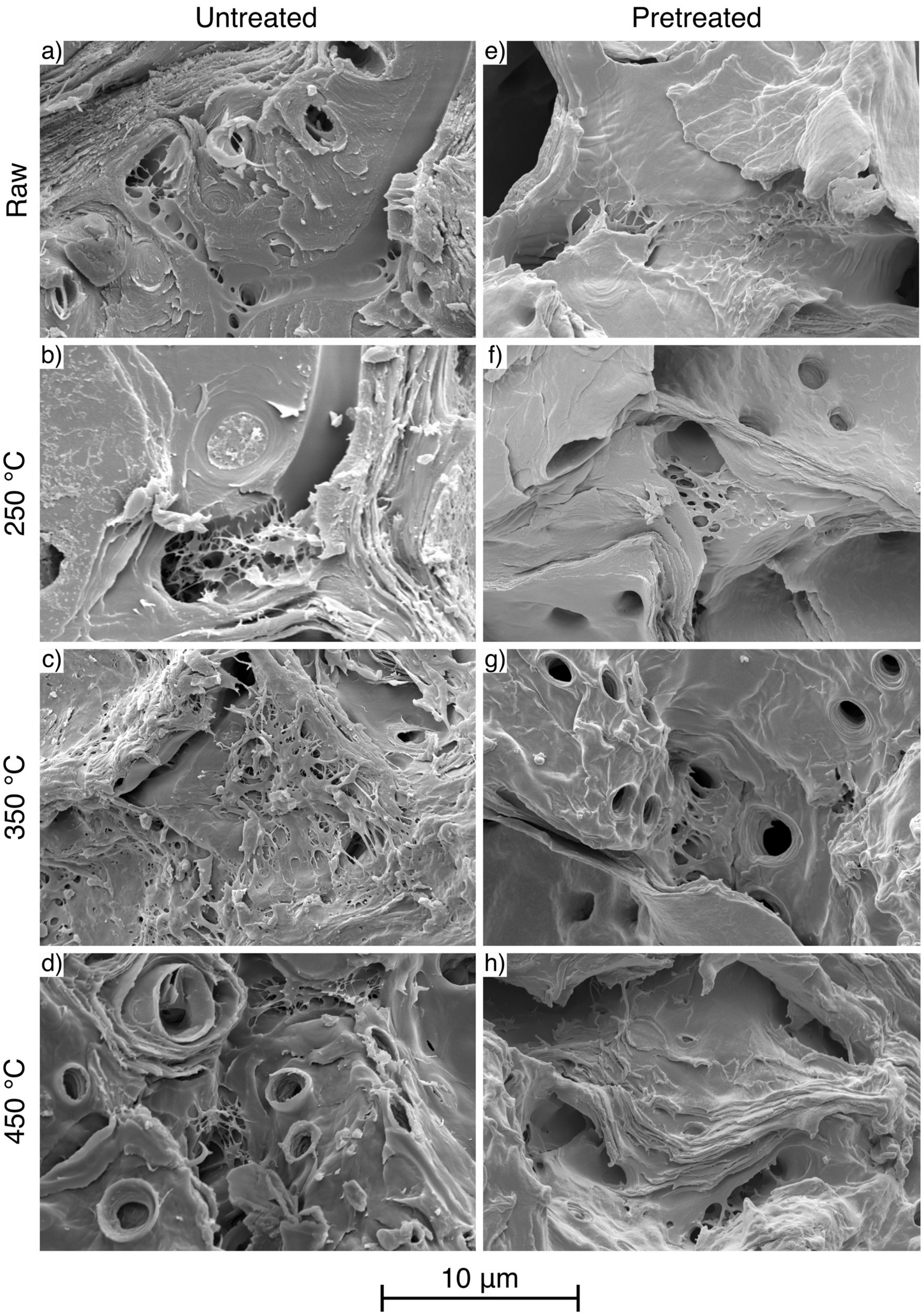

Supplementary Figure 3. Scanning electron micrographs of the apoplast in untreated (a-d) and alkali-pretreated (e-h) walnut shell particles and derived biochars by peak pyrolysis temperature.

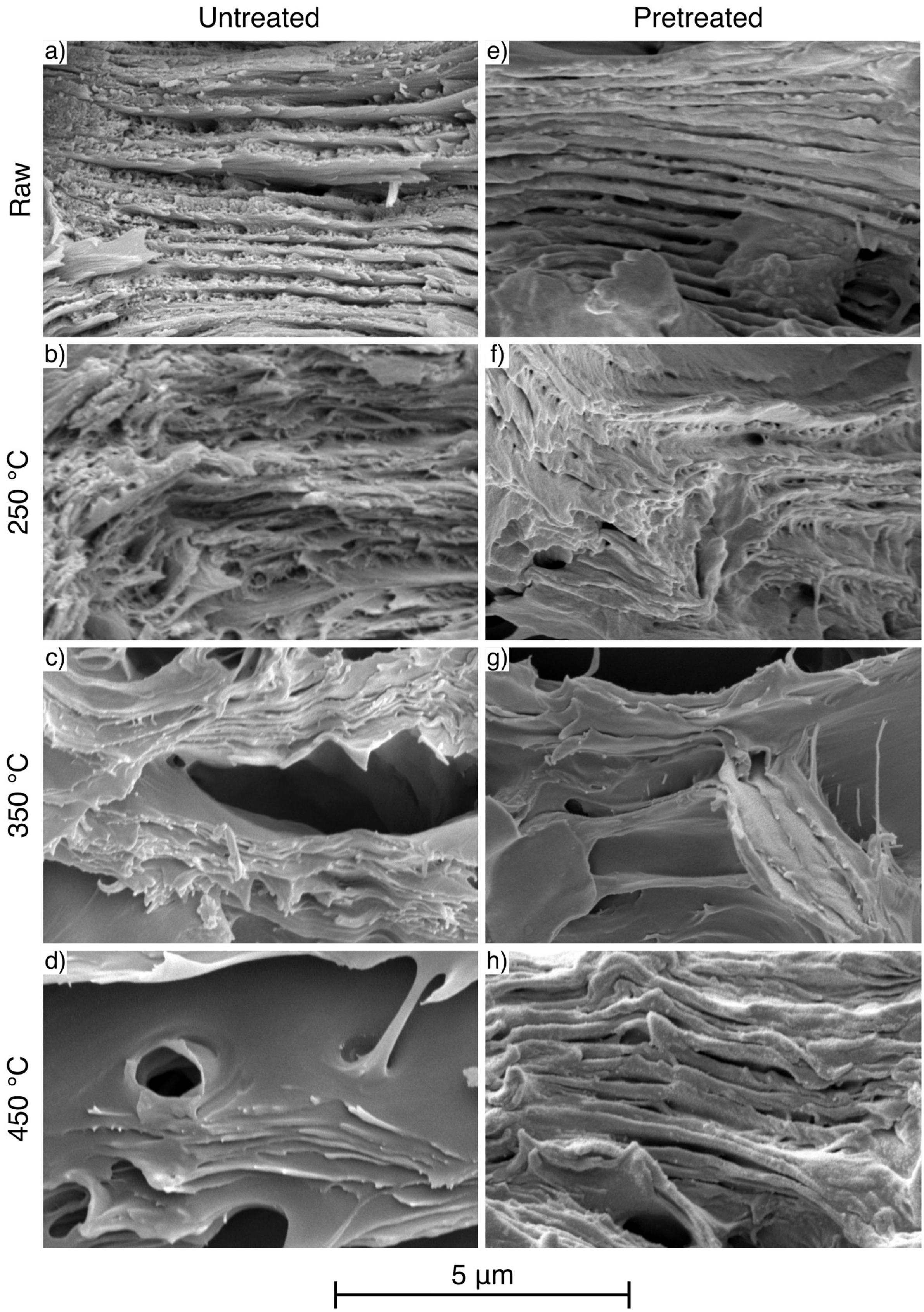

Supplementary Figure 4. Scanning electron micrographs of cell walls in untreated (a-d) and alkali-pretreated (e-h) walnut shell particles and derived biochars by peak pyrolysis temperature.

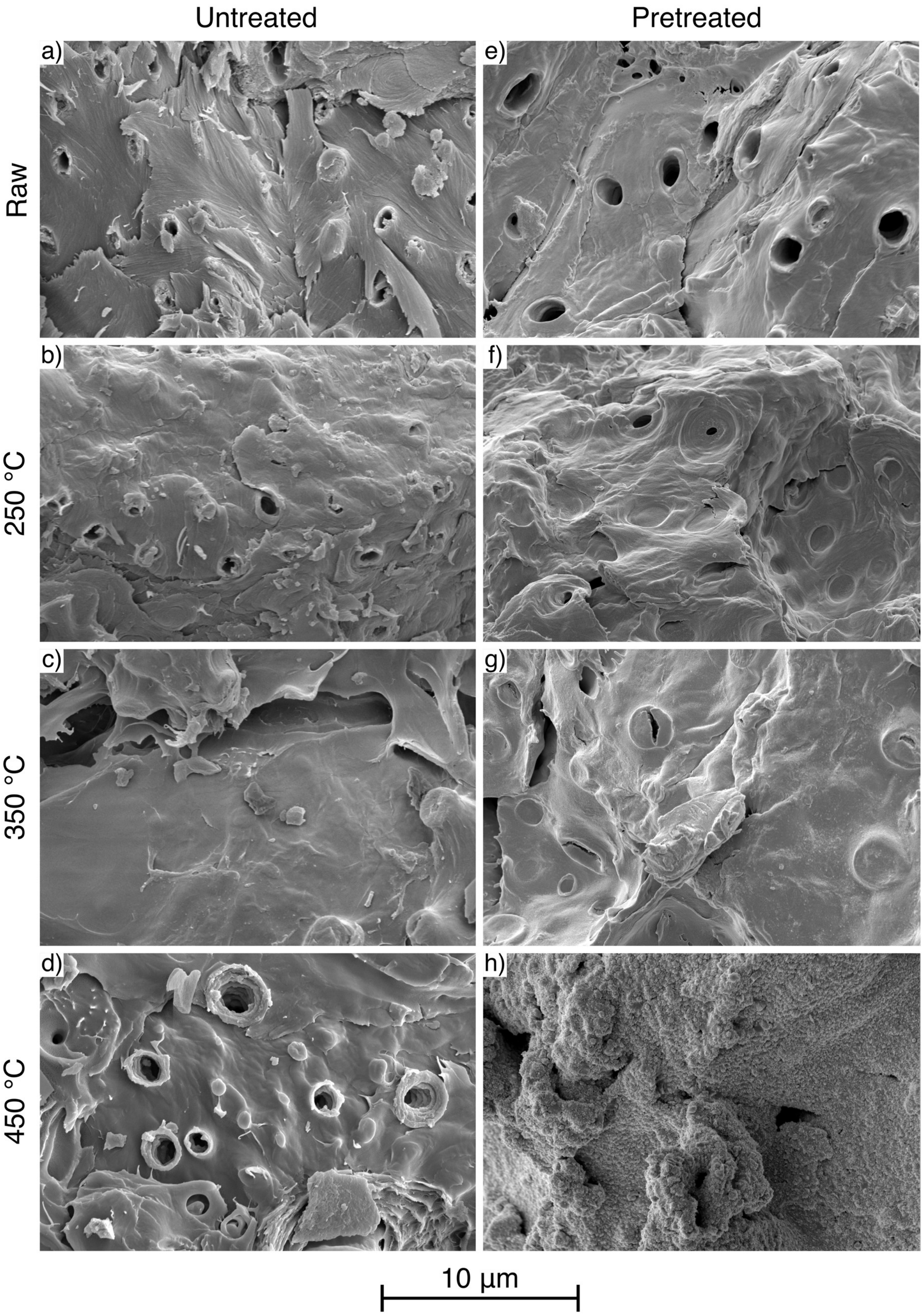

Supplementary Figure 5. Scanning electron micrographs of external cell texture in untreated (a-d) and alkali-pretreated (e-h) walnut shell particles and derived biochars by peak pyrolysis temperature.

Supporting Information: Fady *et al*. Biochar filtration of drug-resistant bacteria and active pharmaceutical ingredients to combat antimicrobial resistance.

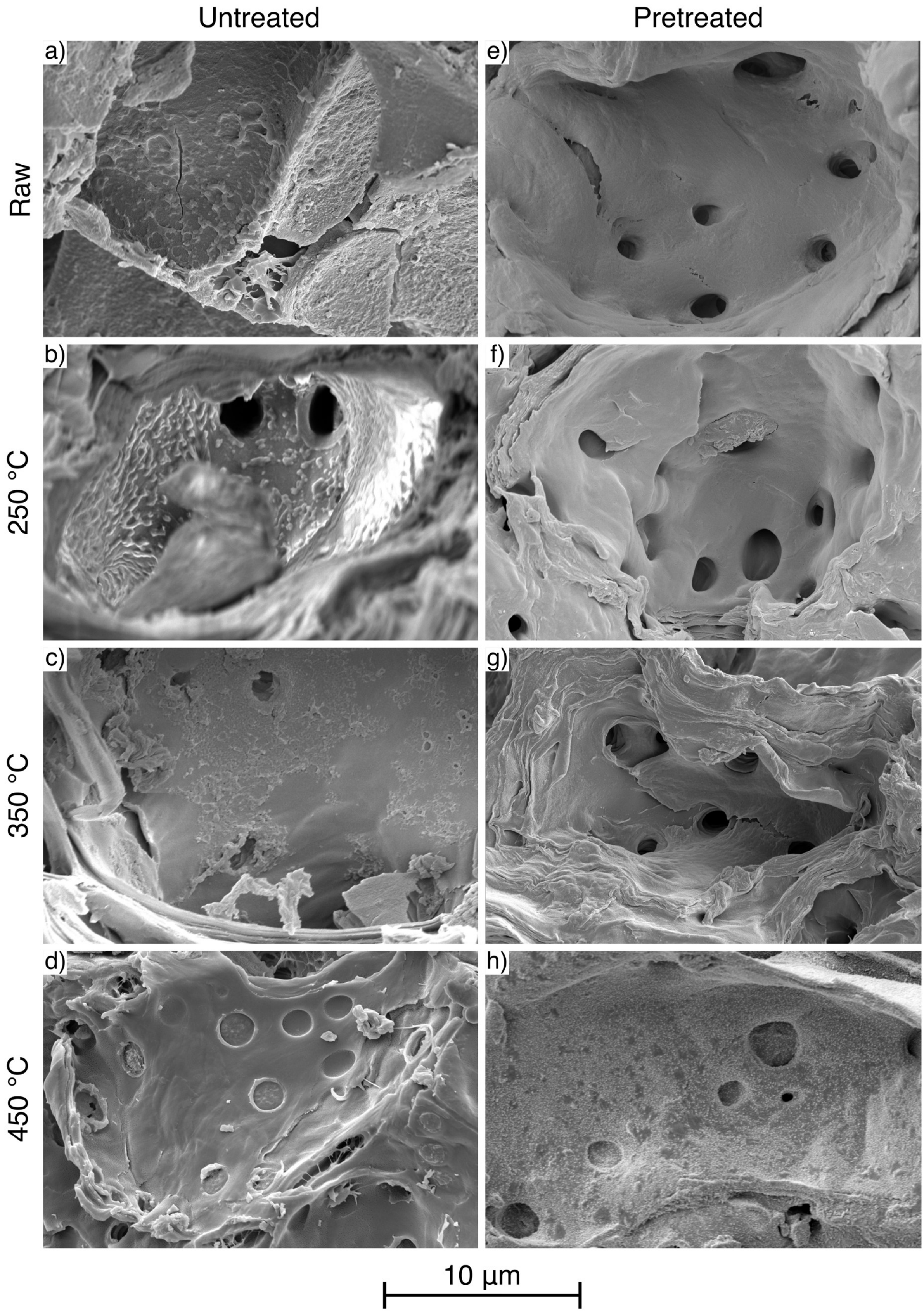

Supplementary Figure 6. Scanning electron micrographs of internal cell texture in untreated (a-d) and alkali-pretreated (e-h) walnut shell particles and derived biochars by peak pyrolysis temperature.
